# Neurobiology and systems biology of stress resilience

**DOI:** 10.1152/physrev.00042.2023

**Published:** 2024-03-14

**Authors:** Raffael Kalisch, Scott J. Russo, Marianne B. Müller

**Affiliations:** ^1^Leibniz Institute for Resilience Research (LIR), Mainz, Germany; ^2^Neuroimaging Center (NIC), Focus Program Translational Neuroscience (FTN), Johannes Gutenberg University Medical Center, Mainz, Germany; ^3^Nash Family Department of Neuroscience, Friedman Brain Institute, Icahn School of Medicine at Mount Sinai, New York, New York, United States; ^4^Brain and Body Research Center, Icahn School of Medicine at Mount Sinai, New York, New York, United States; ^5^Translational Psychiatry, Department of Psychiatry and Psychotherapy, Johannes Gutenberg University Medical Center, Mainz, Germany

**Keywords:** adversity, brain-body, mental health, neuroplasticity, trauma

## Abstract

Stress resilience is the phenomenon that some people maintain their mental health despite exposure to adversity or show only temporary impairments followed by quick recovery. Resilience research attempts to unravel the factors and mechanisms that make resilience possible and to harness its insights for the development of preventative interventions in individuals at risk for acquiring stress-related dysfunctions. Biological resilience research has been lagging behind the psychological and social sciences but has seen a massive surge in recent years. At the same time, progress in this field has been hampered by methodological challenges related to finding suitable operationalizations and study designs, replicating findings, and modeling resilience in animals. We embed a review of behavioral, neuroimaging, neurobiological, and systems biological findings in adults in a critical methods discussion. We find preliminary evidence that hippocampus-based pattern separation and prefrontal-based cognitive control functions protect against the development of pathological fears in the aftermath of singular, event-type stressors [as found in fear-related disorders, including simpler forms of posttraumatic stress disorder (PTSD)] by facilitating the perception of safety. Reward system-based pursuit and savoring of positive reinforcers appear to protect against the development of more generalized dysfunctions of the anxious-depressive spectrum resulting from more severe or longer-lasting stressors (as in depression, generalized or comorbid anxiety, or severe PTSD). Links between preserved functioning of these neural systems under stress and neuroplasticity, immunoregulation, gut microbiome composition, and integrity of the gut barrier and the blood-brain barrier are beginning to emerge. On this basis, avenues for biological interventions are pointed out.

## Graphical abstract

**Figure:**
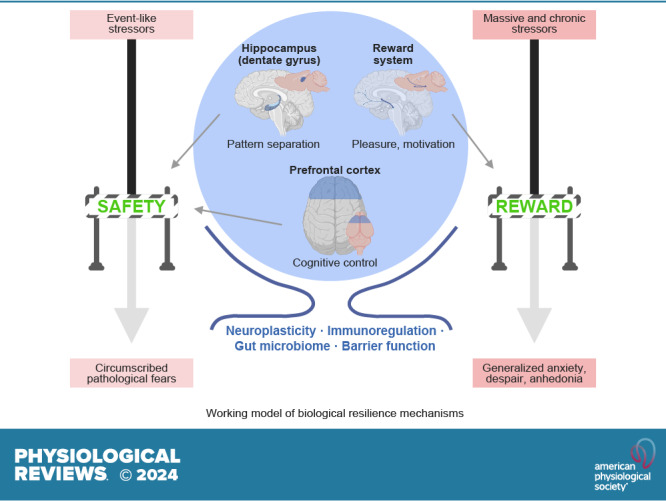

CLINICAL HIGHLIGHTS Stress resilience is the phenomenon that some people maintain their mental health despite exposure to adversity or show only temporary impairments followed by quick recovery. Resilience research attempts to unravel the factors and mechanisms that make resilience possible and to harness its insights for the development of preventative interventions in individuals at risk for acquiring stress-related dysfunctions. Biological resilience research has found preliminary evidence that functions of the hippocampus, the prefrontal cortex, and the reward system support resilience and are in turn supported by factors promoting neuroplasticity, immunoregulation, gut microbiome composition, and integrity of the gut barrier and the blood-brain barrier. This knowledge may open new avenues for biologically based prevention.

## 1. INTRODUCTION

Human life has evolved in a hostile environment full of threats to survival, reproduction, and well-being and characterized by change and unpredictability. As a species, humans have been remarkably successful in dealing with these challenges. As individuals, humans also appear to be masters of adaptation. When confronted with a potential traumatic event (PTE), only a fraction of people develop lasting stress-related mental, physical, or behavioral dysfunctions (1). Adversities of a more temporally extended nature, such as poverty, chronic somatic illness, or enduring social conflicts, also only make a part of their victims sick in the long term (2, 3). Not only are many humans able to withstand stressors, sometimes exposure to stressors can even make humans healthier and happier (4, 5) and more able to deal with future challenges (6–8).

Resilience research tries to understand these remarkable human properties and to use its insights to help individuals whose coping abilities and resources do not match the challenges they are confronted with. Resilience research therefore is both a basic science discipline and an area of applied clinical science, the latter being mainly interested in finding ways to prevent stress-related functional impairments.

### 1.1. Definition

Individual stress resilience has been pragmatically defined as an observable behavioral phenomenon, namely the maintenance or quick recovery of system function during and after periods of adversity, which can be PTEs, longer-term difficult life circumstances, or challenging life transitions (9). Although good system function can be described in many ways, such as in terms of mental health, physical health, psycho-social integration, or attainment of normal developmental outcomes during childhood and adolescence, the vast majority of individual (or “psychological”) resilience studies focus on preserved mental health as the outcome of interest.

The outcome-based definition of resilience as good long-term mental health despite adversity is distinct from a trait-based or a process-based definition. In the early days of resilience research in the 1970s, a frequent assumption was that staying mentally healthy in the face of stressors is determined by some beneficial personality trait, which was equated with resilience (e.g., Refs. 10, 11). Resilience in this view was a natural kind, some type of antinosological entity that one could “have” (or not). Findings that good outcomes are related to a multitude of factors, rather than a single one, and that these can include malleable constructs such as skills, behaviors, or beliefs (e.g., Refs. 12, 13) or also external factors such as social support or cultural influences (e.g., Ref. 14) soon raised doubts about the unitary trait perspective. These doubts were reinforced by observations that people who preserve their mental health in difficult circumstances often develop new strengths, competencies, or perspectives (e.g., Refs. 15–17). These observations included findings of stress inoculation (also known as stress immunization or steeling), the phenomenon that the experience of moderate compared to no or little adversity is associated with better functioning and reduced susceptibility to laboratory stressors later in life (6–8).

Psycho-social resilience researchers have therefore emphasized for some time that good outcomes in many cases probably result from processes of individual change, whereby individuals learn to more effectively and efficiently cope with the hardships they face (16, 18–21). This process-based perspective is also partly inspired by physiological stress research (22–25) and more generally a dynamic systems-theoretical view on the brain/mind (20, 26, 27), which have long pointed out that perturbations that exceed a system’s capacity for coping (that is, stressors) require the system to adapt its mode of operation, by finding new strategies or recruiting additional coping resources. Epigenetic and gene expression studies have more recently supported this perspective and suggested that processes of successful adaptation may also be found on the biological level (e.g., Refs. 28, 29).

**FIGURE 1.**
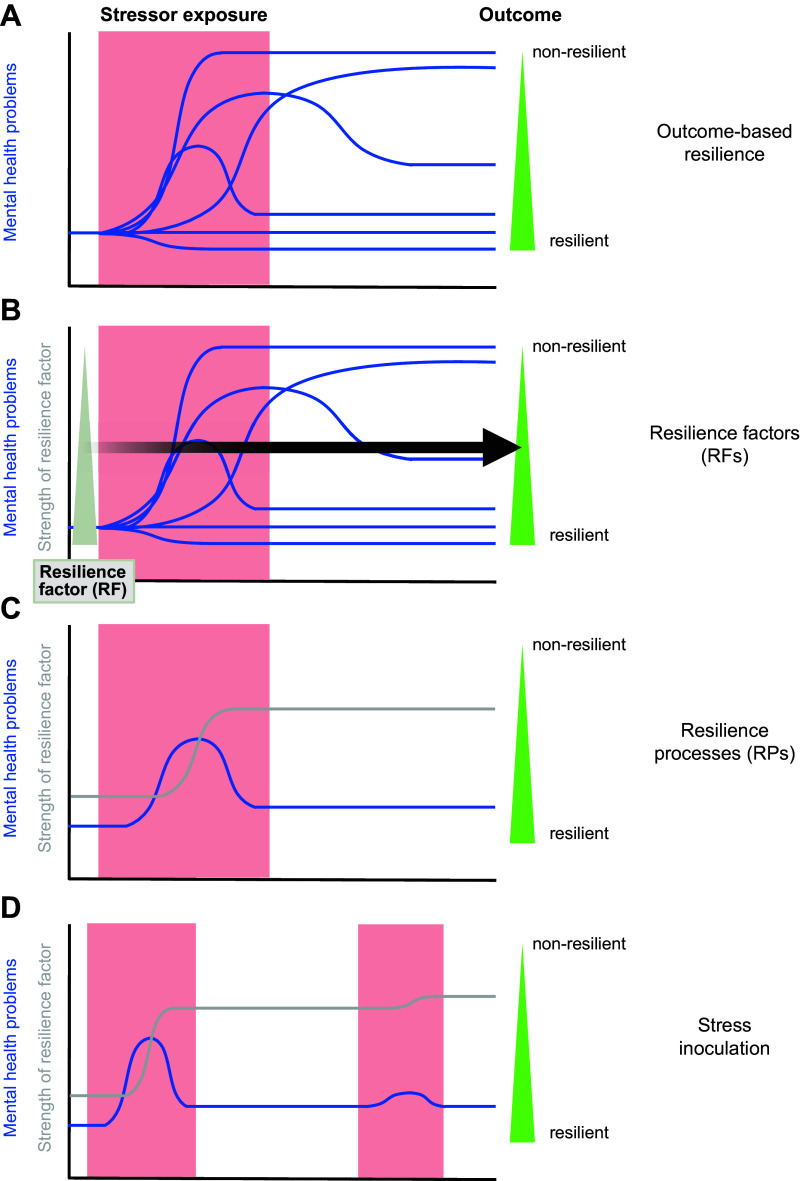
Outcome-based resilience, resilience factors, and resilience processes. *A*: the red box depicts a life episode of substantial stressor exposure, such as a chronic somatic disease, harsh social circumstances, a difficult life transition, or other enduring challenges. Stressful life phases often also follow on a potentially traumatic event (PTE) or other major negative life event. Mental health reactions (blue lines) to comparable stressor exposure can vary greatly between individuals. In an outcome-based framework, individuals with eventual levels of mental health problems (e.g., dysfunctions related to posttraumatic stress, fear, anxiety, or depression symptomatology) that are close to prestressor levels can be classified as more resilient than individuals with lastingly heightened problem levels (*right*). Because, unlike in this hypothetical example, stressor exposure in real life also varies greatly, detailed assessment of individual stressor exposure is as central to determining resilience as assessment of mental health reactions. Otherwise, individuals showing less severe reactions than others only because they are also less exposed may be erroneously classified as resilient (30). *B*: provided appropriate normalization to stressor exposure, one can try to predict good mental health outcomes (fewer mental health problems) from baseline measurements of preexisting resilience factors (RFs), which can be social, psychological, or biological individual-differences variables, stable or modifiable. *C*: a hypothetical example of a comparatively resilient individual who struggles for some time after onset of the stressful life episode to then recover nearly to prestressor levels of mental health problems. Over the course of the coping process, the individual develops higher strength of a modifiable RF (e.g., a better emotion regulation skill), which helps the recovery. Identification of prototypical causal resilience processes (RPs) is a central goal of resilience research. *D*: going through a time of stressor exposure or repeated exposures (first box) in relatively good mental health through an RP (through strengthening of an RF) can make it more likely that one will also go through future adversity (second box) in relatively good mental health (stress inoculation), provided the strengthened RF is suitable to address the future challenge.

The practical problem with adopting a definition of resilience as a process of successful adaptation is that such a definition is hard to operationalize. Processes are by their nature latent and can substantially vary between individuals in timing and quality, which is why they are difficult to observe and to classify. Human resilience researchers are therefore increasingly coming back (9) to a proposal from the early days of resilience research (12) that resilience should be understood and operationalized as an outcome. On this basis, one can then design longitudinal studies in which one assesses participants’ stressor exposure and the associated changes in their mental health, to determine resilient (healthy despite exposure) and non-resilient (dysfunctional, pathological) outcomes. In such studies, social, psychological, or biological variables that prospectively predict resilient outcomes are resilience factors (RFs). RFs may be malleable (such as a particular emotion regulation skill or one’s social support network) but may also include personality traits or other stable features (such as a protective genotype or brain architecture). Provided repeated RF measurement at sufficient temporal resolution, one may also be able to describe resilience processes (RPs), whereby an improvement in one or several malleable RFs prospectively associates with better outcomes (20, 27). For instance, under confrontation with significant adversity, somebody may be forced to more frequently use existing, or to develop new, emotion regulation strategies, with the result that their regulatory skills (a RF) increase and this eventually keeps them from developing lasting mental health problems (see FIGURE 1 for illustration). Interventional designs may target one or several RFs to demonstrate causality.

Hence, the outcome-based definition of resilience allows one to accommodate both a trait and a process perspective. Importantly, however, it does not presume any specific RF or RP as the basis of a resilience definition and, not being theoretically exclusive, is open to new findings and permits researchers pursuing different theories about RFs or RPs to cross talk and to compare their findings against the same benchmark: does my RF or RP generate resilient outcomes (9)? As we see below, the outcome-based definition has also been frequently used in translational animal models of resilience.

### 1.2. Scope

In this review, we focus on biological mechanisms that underlie the resilience of individuals’ mental health to stressor exposure. We restrict our discussion to stress resilience in adults and only consider findings in children and adolescents summarily or where they directly enlighten research in adults, since the timing of adversity with respect to developmental stages and the differential effectiveness of RFs as a function of developmental stage raise a number of intricate questions that complicate the study of biological resilience mechanisms in childhood and adolescence. For similar reasons, we do not discuss resilience in old age. Readers are directed to current reviews (31–36). A final restriction of scope consists of a focus on resilience against dysfunctions or symptoms of the internalizing spectrum, as they are found in the fear-, anxiety-, and mood-related disorders, including among others phobias, panic disorder, generalized anxiety disorder (GAD), posttraumatic stress disorder (PTSD), and major depression, as well as in their subclinical manifestations. The reason for this focus is that the evidence for a role of stressor exposure and stress in disease etiology is by far strongest for these disorders (e.g., Refs. 37–40).

### 1.3. Resilience Mechanisms

Psycho-social resilience research has identified many different RFs, including factors lying outside the individual, such as social support, parental style, or coping-oriented cultural narratives (e.g., Refs. 14, 18, 21, 41). One way to make sense of these findings is to assume that these extraindividual RFs shape the way an individual reacts to stressors (42). For example, the availability of social support may add help-seeking as a reasonable coping strategy to one’s strategy portfolio, or cognitive emotion regulation strategies one has learned as a child from one’s parents by instruction or observation may turn out to be beneficial still in adulthood and their use may be encouraged by a culture that values self-regulation efforts.

**FIGURE 2.**
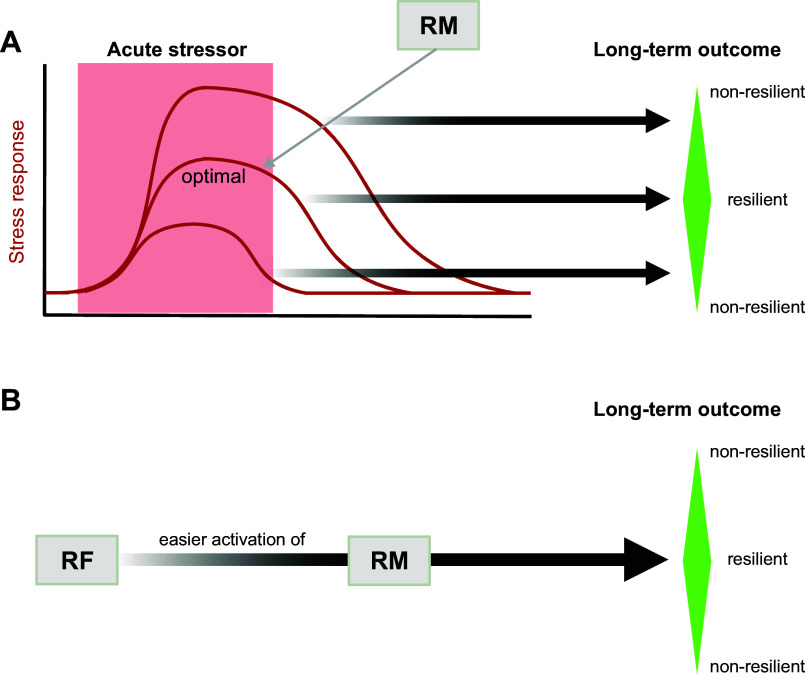
Resilience mechanisms. *A*: schematic of acute stress responses to a short-term stressor. The response shown at *top* overshoots in amplitude and duration, the response shown at *bottom* undershoots, and the response at *middle* is optimal. Typically producing overshooting stress responses increases the likelihood of resource depletion, deleterious allostatic load effects, and eventual lasting mental health problems during and after times of high stressor exposure (*right*). Typically producing undershooting stress responses increases the likelihood that an individual cannot defend themself against threats and pursue their goals and needs and that they will eventually also develop mental dysfunctions when severely challenged over longer times. Resilience mechanisms (RMs) are mental, bodily, or somatic operations that regulate acute stress responses to optimal levels and thereby increase the likelihood of good mental health outcomes despite adversity (= resilience). *B*: resilience factors (RFs) are associated with resilient outcomes (*right*) because they make the activation of RMs during acute stress situations more likely.

Resilience mechanisms (RMs) then are mental, bodily, or behavioral activities that occur when an individual is acutely confronted with a stressor and that make it likelier that the individual will survive confrontation with the current and future stressors in good mental health (9, 24) (FIGURE 2*A*). RFs are predispositions or conditions that make the activation of these RMs more likely (9) (FIGURE 2*B*). In the example of help-seeking, if a concrete act of help-seeking is the mechanism by which someone copes in a given situation, successfully and in a way that does not undermine future coping (that is, help-seeking is the RM), then one’s ability and willingness (tendency) to seek help are intraindividual RFs that predispose the individual to employ this strategy. The strength of intraindividual RFs in turn may depend on other intraindividual RFs (e.g., communication skills, personal value system, functionality of the brain circuitry mediating help-seeking, genetic background) and also extraindividual RFs (e.g., availability of help/social support, cultural value system). RPs (FIGURE 1*C*) occur when there is a change in this dispositional landscape, for instance, when successful coping via help-seeking reinforces an individual’s help-seeking tendencies or motivates somebody to invest more in their social embeddedness, to make social support more easily available in future stressor situations. Another example was given above for the case of emotion regulation skills improving during adversity and facilitating coping. (Note that not all challenges may require, or induce, such adaptation processes, especially when they can be easily overcome with existing resources.) Thus, reacting to stressor exposure involves different timescales. On a short timescale, RMs are activated to acutely cope with present stressors in an optimal fashion (FIGURE 2*A*); RM activation on this timescale is affected by the architecture of one’s RFs (FIGURE 2*B*). On the longer timescale of RPs, a person’s RF architecture is sometimes changed, thereby affecting future coping (FIGURE 1*C*). Biological resilience research is concerned with understanding *1*) RMs, *2*) intraindividual RFs, and *3*) the individual capacity for long-term change (that is, for the occurrence of RPs). The latter is system property that must involve learning and memory functions or other forms of plasticity and, as far as it exhibits individual differences, may in itself be considered an RF (and perhaps a crucial one).

A guiding principle in the investigation of biological RFs and RMs is that resilience is unlikely to result from some extraordinary or “superhuman” capacities. Rather, resilience should be rooted in the good and normal functioning of the body and brain systems that are called into battle when humans encounter stressors. This obtains from the high prevalence of resilience, as a frequent outcome of even severe adversity, and the evolutionary success of the human species. Another useful consideration is that not all RMs may protect against all types of stress-induced dysfunction (some may be dysfunction specific, some general) and even general RMs may not protect against all types of stressor or work in all types of stressor-exposed populations (some may be stressor and/or population specific, some may be global) (30). Arguably, the most interesting targets for the development of preventive interventions would be general and, more so, global RMs.

## 2. FUNCTIONAL-MECHANISTIC THEORIES OF RESILIENCE

### 2.1. Stress and Optimal Stress Response Regulation

Stress is a reaction to stimuli or situations perceived as threats to the organism (25, 43, 44). Stress responses involve changes in attention and cognition (attentional focusing, information gathering, stressor appraisal, planning for coping, cognitive coping), affective experience (feelings of nervousness, fear, anxiety, anger, sadness), behavior (active coping), and underlying central nervous system and peripheral physiology [including activation of the brain’s stress network, the sympathoadrenal medullary (SAM) system, and the hypothalamus-pituitary-adrenal gland (HPA) axis]. The function of stress is to adapt to a dynamic environment and thereby to preserve the organism, its well-being, and its reproductive abilities (23, 25, 44, 45). Thus, stress is primarily beneficial. Because stress is also resource consuming, stress can become maladaptive and damaging and eventually cause disease when exaggerated or chronic (23) (FIGURE 2*A*).

The hostile nature of life and the adaptive function of stress imply that resilience cannot result from stress avoidance alone. Rather, stress responses should be optimized to fulfill their protective role as much as necessary, but not more (30). Stress-related resource exhaustion and dysfunction are less likely when individuals manage to mount stress responses whose quality and magnitude are appropriate for the situation (proper strategy selection and fine-tuning) and do not extend to safe aspects of a threatening situation, terminate when the threat is over, and disappear altogether when the former threat no longer is one (threat-safety discrimination) (30) (FIGURE 2*A*).

On an abstract level, biological systems capable of behaving in this way need a balance of excitatory and inhibitory functions, the former ensuring resource mobilization to threats and the latter preventing the system from spiraling into uncontrolled excitation.

These general considerations based on a functional analysis of stress place the optimized regulation of stress responses at the heart of theorizing about RMs. In this perspective, there is no single coping strategy (e.g., help-seeking) that will guarantee preservation of mental health, but success comes from efficient resource deployment, which may involve situationally coherent strategy selection, switching to alternative strategies when necessary, and fine-tuning of the magnitude and duration of the response (30).

### 2.2. Flexibility- and Appraisal-Based Resilience Mechanisms

**FIGURE 3.**
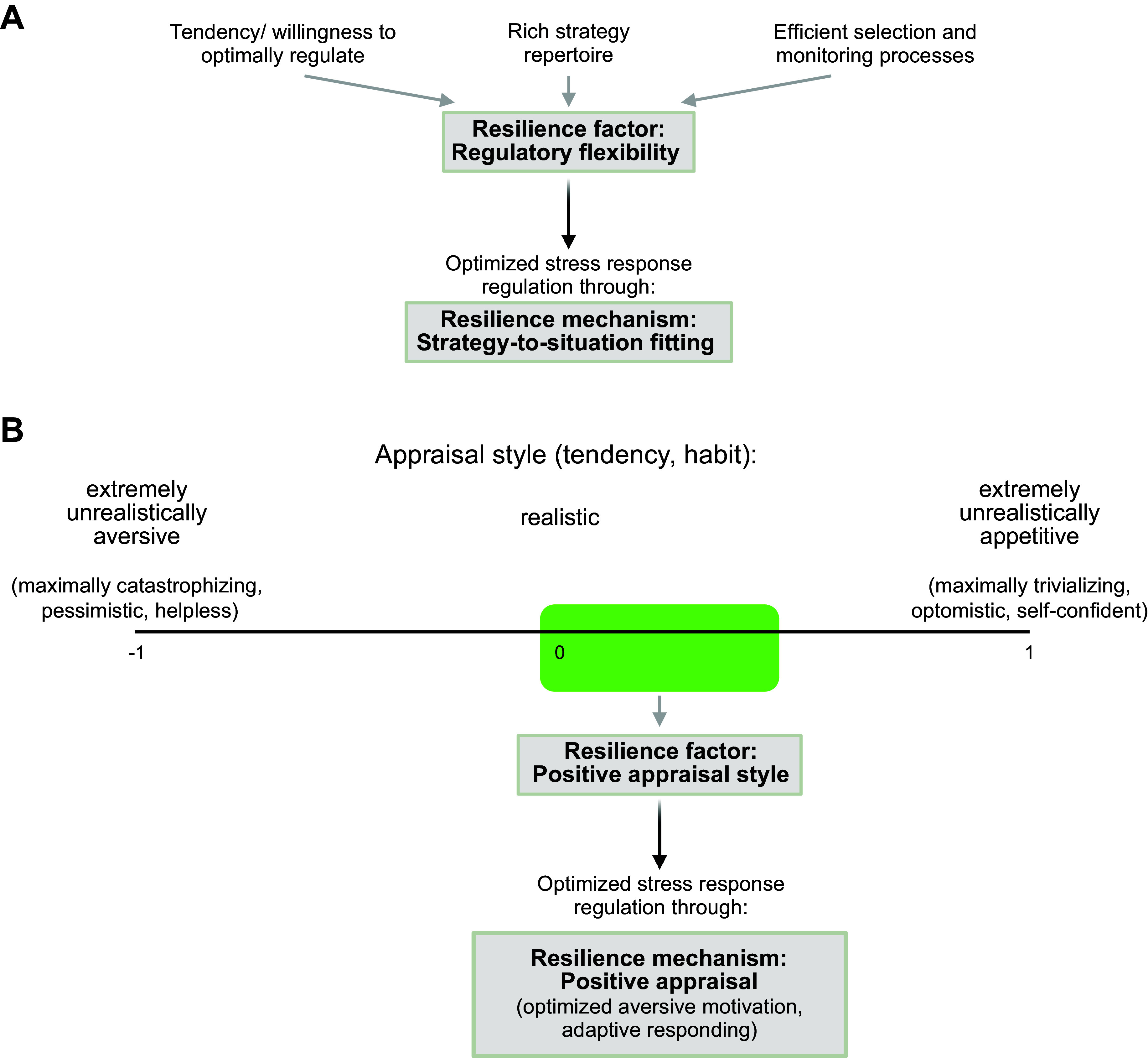
Functional-mechanistic theories of resilience. *A*: regulatory flexibility theory posits that a combination of individual-differences factors (*top*) generates a tendency or predisposition (the resilience factor) to flexibly choose regulation strategies as a function of their fitting situational demands (the resilience mechanism). Stress responses thus become optimal, on average. *B*: positive appraisal style theory posits that a tendency or predisposition (the resilience factor) to appraise stressors in a mildly unrealistically positive fashion (the resilience mechanism) on average optimizes aversive motivation in stressor situations to levels that guarantee sufficient resource mobilization of resources while avoiding extreme, including inflexible, responding.

These considerations also relativize the example of effective help-seeking as an RM and raise the question of whether RMs are rather to be found in some superordinate regulatory function or functions. This thinking ties in with a class of psychological resilience theories revolving around the idea of regulatory flexibility (46–51), starting from the conjecture that not all coping strategies are always helpful. Help-seeking does not help when no support is available, and depending on who is available as a helper it may create unwanted dependencies; frequent recurrence to help-seeking may also undermine one’s agency. Fighting an aggressor might be a sensible approach when chances of winning are high, but otherwise flight, submission, or negotiation might be preferable action paths. Distraction may be good to save resources when nothing can be done (you are sitting in a plane flying through a storm) but not when action is necessary (you are the pilot). Flexibility theories therefore emphasize the importance of good strategy-situation fit and search for RMs in the neurocognitive processes that ensure proper, contextually sensitive strategy selection rather than only in the processes serving to execute any particular strategy. These superordinate processes include recognizing situation characteristics, identifying and implementing the best matching strategy, and monitoring regulation success, in order to be ready to switch (47, 49, 50, 52). Having a rich repertoire of strategies and efficient selection and monitoring mechanisms at one’s disposal and being inclined to employ them in stressful situations are key individual RFs, that is, they make flexible regulation in stressful situations more likely (FIGURE 3*A*).

Positive appraisal style theory of resilience (30, 42) conjectures that such smart, flexible responding to stressors is compromised or impossible when one is overwhelmed by aversive motivation; by contrast, appraising (evaluating, interpreting) stressors in a realistic to mildly unrealistically positive way allows one to mobilize the necessary energy for responding while at the same time avoiding inflexible overreactions. Thus, appraisal theory places key RMs upstream to situation-fit strategy selection in the various conscious/verbal and nonconscious/nonverbal stressor appraisal processes that determine one’s degree of aversive motivation. The key RF is when one’s individual appraisal tendency, or style, is biased toward mild optimism (rather than pessimism) on the appraisal dimension of threat probability, mild trivialization (rather than catastrophizing) on the appraisal dimension of threat magnitude, and mild overconfidence (rather than helplessness) on the appraisal dimension of controllability, or power (FIGURE 3*B*).

### 2.3. Flexibility- and Appraisal-Based Resilience Processes

Both flexibility- and appraisal-based theories highlight the role of learning from experience, or experience-based plasticity. In flexibility theory, individuals take into account success or failure of strategy applications in past contexts to determine the likely optimal strategy for the current context (49). In positive appraisal style theory, the key RP consists in someone developing a more positive appraisal style based on experiences of safety or successful coping (26, 30). Thus, both flexible selection and positive appraisal tendencies (or more generally speaking, good stress regulation ability) are considered malleable RFs, which to strengthen over confrontation with adversity is the crucial process of adaptation (an RP) (30).

Hence, successful coping or stress regulation depends on past positive experiences and is the source of new positive experiences (53). Furthermore, insofar as acute stress states hinder the formation and consolidation of new and appropriately contextualized memories, the updating of older memories, and the transfer and generalization of these new memory traces to other situations (54) and insofar as stress states also specifically have a detrimental effect on safety memories (55), good stress regulation provides not only beneficial memory contents but also optimal conditions for the long-term storage and retrieval of these desirable memories.

Taken together, these considerations suggest a bidirectional positive interaction between optimal stress response regulation and optimal long-term memory processes.

## 3. BEHAVIORAL PARADIGMS TO INVESTIGATE BIOLOGICAL RESILIENCE MECHANISMS IN HUMANS

**FIGURE 4.**
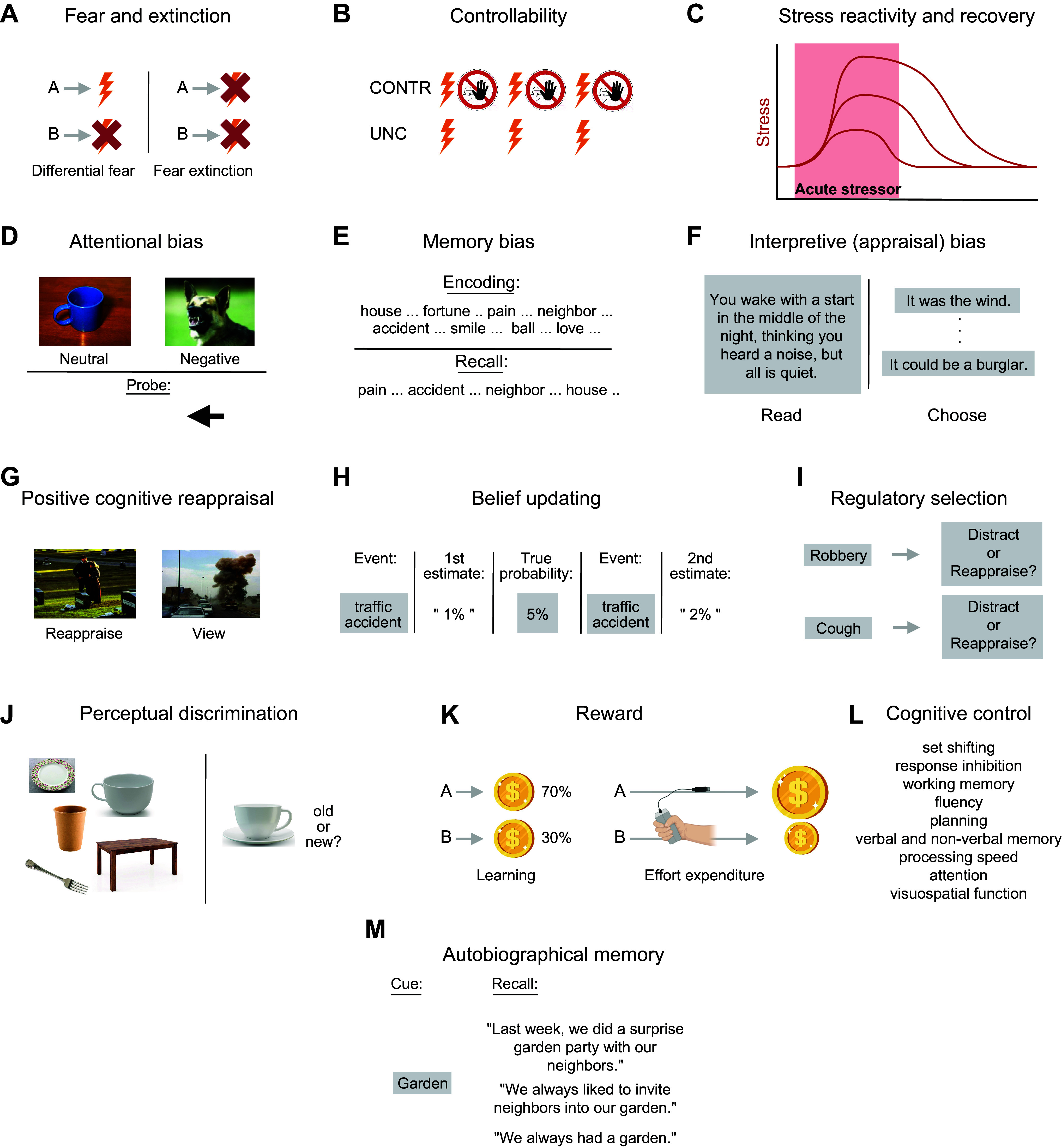
Behavioral paradigms used in resilience research. *A*: differential fear paradigms present instructed or conditioned stimuli that predict either threat (*A*), such as pain, or safety (*B*). Later repeated presentation of *A* in the absence of an aversive outcome leads to fear extinction, whereby *A* now also signals safety. *B*: controllability paradigms apply identical amounts of aversive stimuli in a condition or group where the participant can (CONTR) or cannot (UNC) stop, or escape from, the stimulation*. C*: stress reactivity and recovery paradigms test the increase and decrease of attentional-cognitive, affective, behavioral, or physiological (e.g., cortisol) responses to more complex stressors (e.g., aversive film clips, imagery, social performance pressure). *D*: the dot probe task tests attentional stress reactivity (attentional bias) by asking participants to quickly indicate features of a cue presented at the location of either a neutral or a negative preceding stimulus. *E*: episodic learning and short-term memory paradigms test whether individuals are biased toward recalling negative over neutral or positive stimuli. *F*: to test interpretive (appraisal) biases, ambiguous scenarios are presented, and participants then have to choose among several plausible outcomes of different valences. *G*: positive cognitive reappraisal tasks test the ability to generate positive reappraisals of negative situations, relative to a condition without reappraisal effort (e.g., just view). *H*: in belief updating tasks, participants give a first probability estimate for a negative life event and then receive undesirable corrective information, after which the extent of them integrating that information is measured in a second estimate. *I*: in regulatory selection tasks, the tendency to regulate emotion with either reappraisal or distraction is measured as a function of negative stimulus intensity [high (*top*) vs. low (*bottom*)]. *J*: to assess perceptual discrimination (pattern separation), mnemonic similarity tasks first show different objects or scenes to later see whether participants tell lures (new objects similar to old objects) from truly old objects and new objects. *K*: in probabilistic reward learning tasks (*left*), participants learn through trial and error that 1 of 2 available stimuli (*A*) is more frequently followed by reward (e.g., money) than the other (*B*). In effort for reward paradigms (*right*), investing more effort (e.g., gripping harder) is rewarded by higher gains, such that willingness to invest can be assessed. *L*: cognitive control functions are assessed with many different tasks, sometimes in large neuropsychological test batteries. *M*: autobiographical memory paradigms give cues to recall past life episodes, which are then judged on detail and specificity (e.g., example at *top*, vs. repeated or gistlike recollections at *middle* and *bottom*).

The preceding functional-mechanistic analysis provides useful guidance for research into biological mechanisms of resilience. Because strategy selection and appraisal are functions of the brain, key biological RMs are to be sought in the brain circuits that implement these functions. This is not as trivial as it might appear, because it suggests that findings about resilience-conducive biological processes in the brain’s bodily environment ultimately require an explanation in terms of how they affect specific central nervous system processes if one is to make mechanistic sense of them. Furthermore, experimental psychology and cognitive neuroscience have developed behavioral paradigms, some translatable to animal models, that allow one to examine individual differences in these brain-based mechanisms, test whether these differences are related to resilience (qualify as RFs), and study their neural underpinnings and their central and peripheral biological determinants. See FIGURE 4 for an overview.

### 3.1. Detecting, Discriminating, and Remembering Threat and Safety

#### 3.1.1. Instructed and Pavlovian fear paradigms.

Of highest theoretical interest are threat-safety discrimination paradigms that present both threatening and safe stimuli or situations, such that both an individual’s ability to produce an aversive response when required (excitatory responding) and their ability to abstain from responding when not required (response inhibition) are tested. In differential fear paradigms, a stimulus, which can be a discrete cue or a contextlike configuration, is assigned threat value by virtue of an experimental instruction that it will or may be accompanied by an undesirable other stimulus or outcome, e.g., a painful electric stimulus, an annoying sound, or an unpleasant picture (“instructed fear”), or by directly providing the stimulus-outcome contingency experience (Pavlovian fear conditioning). Critically, a second stimulus is by instruction or experience safe, that is, predicts the absence of the unpleasant outcome (FIGURE 4*A*). In the language of associative learning theory (56), the outcome is the unconditioned stimulus (US), the threat stimulus is the CS+ (conditioned stimulus paired with the US), and the safety stimulus is the CS−. In the language of appraisal theory (43, 57, 58), the US prediction carried by the CS+ conforms to the appraisal of the CS+ as a stressor that signals a threat with a certain probability and magnitude. Fear reactions can be measured through self-report of affect, US expectancy, or CS-US contingency knowledge or with a variety of physiological indexes, such as skin conductance, heart rate, facial muscle contraction, or potentiation of muscular reflexes to a different, sudden, and intense stimulus (startle probe).

Testing for successful excitation to the CS+ (to confirm that the threat response is neither blunted nor exaggerated) and inhibition to the CS− (to confirm discrimination of safety from threat) can be done acutely during conditioning or in a later experimental session. Delayed tests often use a range of additional so-called generalization stimuli (GSs) that are presented intermingled with the CS+ and the CS− and are built to establish a gradient of perceptual or conceptual similarity between the CS+ and the CS−. In this test, a steeper decline of conditioned responding (CR) from the CS+ across increasingly less CS+-similar and more CS−-similar GSs toward the CS− indicates better discrimination (59). Delayed tests assess the strength and accessibility of the safety memory formed during the acute safety learning phase of the paradigm.

#### 3.1.2. Fear extinction paradigms.

A variant of safety learning obtains when the (instructed or experienced) CS+ is repeatedly and consistently presented in the absence of the US, such that it should ideally be reappraised as safe (FIGURE 4*A*). Fear extinction, too, can be tested acutely during extinction learning or later. The return of the CR that is often observed when a successfully extinguished CS+ is presented again in a later test session (60) shows that safety learning success does not translate one-to-one into successful safety memory consolidation and retrieval; hence, safety learning and memory functions are governed by (partly) different neural mechanisms (61) and can be separately studied in the extinction paradigm. This is relevant for the investigation of long-term adaptive RPs, which by definition require some form of positive long-term memory (see sects. 1.3 and 2.3).

#### 3.1.3. US deflation and controllability paradigms.

Although most fear paradigms manipulate the likelihood by which a threat occurs (threat probability dimension of appraisal), manipulations of the threat magnitude dimension of appraisal are implemented in US deflation paradigms, where after conditioning the US is presented in reduced intensity or in combination with safety information, to then test to what extent the CR to a later CS+ presentation is also reduced (62, 63). Manipulations of the control dimension of appraisal (sect. 2.2) are implemented in various forms of controllability paradigms. Here, the experimental subject is given the opportunity to escape from, or end, an aversive stimulation, and this is compared to another experimental condition or group where the amount of aversive stimulation received is identical but not under control of the subject (64, 65) (FIGURE 4*B*). Performance testing in the latter types of safety learning paradigms often involves delayed presentation of the original threat stimulus but also of other types of stressors and thereby also allows quantifying to what extent safety memories generalize to different situations. Perceptions of controllability can also be induced by instruction (e.g., Refs. 53, 66).

#### 3.1.4. Stress reactivity and recovery paradigms.

Yet another form of safety consists in the termination of threat. Acute safety detection after a stressor can be inferred from stress recovery, that is, the speed of the decline of the excitatory response to the acute stressor, and can be studied alongside stress reactivity, that is, the amplitude of the excitatory response (67). Reactivity and recovery paradigms typically use temporally more extended and complex stressors compared with instructed or Pavlovian fear paradigms [e.g., aversive film clips, (68); instructed aversive imagery (69)] and often comprise a social-evaluative threat (e.g., anticipating to give, or giving, a speech in front of a critical audience, or performing a math task under time pressure while receiving negative feedback) (70, 71) (FIGURE 4*C*). They may employ different response measures (attentional-cognitive, affective, behavioral, physiological). Unlike simple CSs, they usually activate not only the SAM system but also the HPA axis.

Another class of reactivity paradigms use less severely negatively valenced stimuli that do not evoke strong physiological reactions, such as pictures of angry or sad faces or spiders or snakes, as experimentally easy-to-handle proxies of threat. The most popular of these paradigms is the dot probe task (72), where a negative and a neutral stimulus are briefly shown on a computer screen, followed by a visual probe that appears at the location of one of the former stimuli. The probe itself comes in two variants (e.g., a left- or right-pointing arrow), and participants have to indicate which of the two probes they see as quickly as possible by pressing the corresponding button on a keypad (FIGURE 4*D*). Shorter reaction times to, or quicker or longer visual fixation of, the probe appearing behind the negative stimulus indicate an attentional bias (enhanced attentional reactivity, or hypervigilance) to threat (73). The paradigm does not permit investigation of recovery.

Reactions to stressors depend on their appraisal, and stressor appraisal is heavily influenced by memories of past experiences with the same or comparable stressors. The ways in which threat and safety are encoded into, and retrieved from, episodic memory are therefore important to understanding stressor reactivity. At the same time, they represent another dimension of stressor reactivity: high reactivity is likely to involve preferential learning and retrieval of negative episodic information, whereas low reactivity is likely to involve better learning and retrieval of positive information. In typical episodic learning and short-term memory paradigms, participants are first shown a range of stimuli (e.g., words, pictures) that can be neutral or emotional (positive, generally negative, threatening, self-referential) and that they are asked to process either deeply (by thinking about them or relating them to each other) or superficially (by focusing on, and responding to, some perceptual features). Retrieval is later operationalized through free recall, where participants are asked to recollect as many stimuli as possible in any order and the fraction of recollected stimuli is counted and compared between valences (FIGURE 4*E*). Other test methods include recall cued by word stems or decision tasks where participants have to indicate whether they recognize or not a presented stimulus as previously shown (74, 75). These paradigms are also not suitable to investigating recovery.

#### 3.1.5. Paradigms targeting explicit and declarative processes.

Safety is a relative construct that only exists by reference to a threat (76, 77) and is eventually determined not by some objective property of a situation but by its subjective appraisal. In real life, inferring safety from environmental predictors of reduced danger (as in discrimination, extinction, deflation, recovery) or one’s own behavioral coping potential (as in controllability) can be made difficult by the complexity, ambiguity, and unpredictability of situations. In such multidimensional situations (as emulated in some stress reactivity paradigm), to eventually appraise a situation as safe humans presumably need to make more cognitive efforts and to rely more strongly on higher-order (conscious and language based) cognitive process than may be the case in simple fear paradigms.

The individual tendency to generate such relatively explicit safety appraisals can be directly tested, for instance, by having participants read sentences describing ambiguous scenarios (“You wake with a start in the middle of the night, thinking you heard a noise, but all is quiet”) and then asking them to choose one of several presented explanations, which can range from not threatening (“it was the wind”) to highly threatening (e.g., “it could be a burglar”) (FIGURE 4*F*). Here, preferential choice of nonthreatening explanations indicates an interpretive (appraisal) bias toward safety (78).

The individual ability, or capacity, to explicitly generate positive appraisals can be tested in positive cognitive reappraisal tasks, where participants are instructed to see a situation (e.g., a negative scenario presented in a picture or a text vignette or the anticipation of receiving an aversive stimulus) in a positive light in order to downregulate their emotional response to the situation (FIGURE 4*G*). Subjective-affective or physiological response indexes in the reappraisal condition are then compared to a condition in which participants are requested to react naturally, that is, without a regulatory effort, to the situation (79).

As for instance in extinction, positively reappraising a situation through explicit-declarative efforts can lastingly change the perception of, or reaction to, a stimulus or situation (80), that is, leave a safety memory trace. It may also generate memories of cognitive mastery (the knowledge that one is able to cope with, or regulate, unpleasant emotional states), that is, a control-related form of safety that is associated with expectancies about future regulation success and may thus be another basis for future facilitated stress response regulation (81). Successful cognitive emotion regulation through other strategies (such as distraction or expressive suppression) may also optimize future stress responding in the case where they generate safety or mastery memories (30).

Explicit safety learning also takes place in belief-updating paradigms, where participants first have to give an estimate of the probability of incurring a given negative life event, such as divorce or a cancer diagnosis (another way of assessing interpretive bias, on the probability dimension of threat appraisal), and are then presented with correct, scientifically based probability information. If this information deviates from the participant’s estimate, there is an expectation violation that should lead to an adjustment of one’s estimate that can be tested in another presentation of the same life event shortly afterward (82) (FIGURE 4*H*). Safety learning is evidenced by the participant updating their estimate toward a reduced likelihood in the case of better-than-expected information. Belief updating is a form of declarative learning.

### 3.2. Selecting the Right Strategy

Whereas the paradigms discussed so far investigate pathways to stress responses that are optimized in their magnitude or duration, another aspect of stress response optimization lies in the selection of the situationally most appropriate type of coping response. Based on evidence that a coping strategy of distraction is more effective in the short term than a strategy of positive cognitive reappraisal in limiting aversive responses when the aversive situation is highly intense and that the opposite applies to aversive situations of low intensity, Sheppes (83) has developed a regulatory selection task where a participant is presented with a negative emotional stimulus of either high or low intensity and then has to choose between using either distraction or reappraisal (FIGURE 4*I*). Healthy individuals consistently prefer distraction over reappraisal in high-intensity situations and reappraisal over distraction in low-intensity situations, indicating an ability to flexibly match strategy selection to situational demands.

### 3.3. Supporting the Aversive System

The tasks used to test optimal stress response regulation activate excitatory processes in the aversive, or stress, system, that are responsible for stress response generation, and it can be assumed that this system comprises built-in brakes, that is, intrinsic inhibitory processes that contain overexcitation (as has been shown for instance for the negative feedback that released cortisol exerts on HPA axis activity) (84). Inhibition may, however, also be conferred by other neural systems that interact with the aversive system in shaping stress responses.

#### 3.3.1. Perceptual discrimination (pattern separation) paradigms.

The ability to discriminate between threat and safety is, at least in part, driven by the ability to discriminate between the perceptual features of threat and safety stimuli (85). Nonemotional separation of sensory patterns can be assessed with the mnemonic similarity task (86), where first a series of objects or scenes are shown to participants, coupled with the requirement to judge them according to some irrelevant feature (e.g., indoor or outdoor), and then a surprise recognition test is conducted during which participants are given previously seen (old) and new stimuli (FIGURE 4*J*). Some of the new stimuli (lures) are similar to the old ones, and better discrimination between old and lure stimuli (better pattern separation) is associated with better threat-safety discrimination in the delayed gradient-based discrimination test (87, 88), though not in simple differential fear conditioning (89), where perceptual demands are modest. Good pattern separation in the mnemonic similarity task may also be related to easier discrimination of threatening and safe contexts (90). Discrimination between similar negative items is generally poorer than discrimination between similar neutral items, indicating emotional costs of pattern separation (91).

#### 3.3.2. Reward (appetitive) paradigms.

Observations that reward learning to a CS delays subsequent fear learning to the same CS and vice versa (92–95), that fear extinction learning and memory are carried by a dopaminergic circuitry that overlaps with the reward learning and memory circuitry (26, 96), that attention to positive stimuli inhibits processing of negative stimuli and vice versa (97), that positive and negative affective states occur in an anticorrelated fashion (98), and that positive affective states dampen acute stress responses, including at a neural level (99), together strongly establish the reward system as an antagonist of the aversive system. This makes paradigms probing Pavlovian or instrumental reward learning, decision-making (choice) tasks involving positive options, tasks measuring the effort spent on obtaining rewards, and positive emotion stimulation in general (99–101) interesting for resilience research (FIGURE 4*K*).

#### 3.3.3. Cognitive control paradigms.

Aversive system inhibition can also be afforded by higher-order executive functions that can be used to exert control over aversive stimulus processing and behavioral responses (102). Cognitive control systems are demonstrably involved in positive cognitive reappraisal (103, 104), strategy selection (83), and more generally in any task requiring effortful and declarative processing of emotional information. Executive functions can be subdivided into the domains of inhibition [deliberate suppression of salient stimulus processing (selective attention) or of prepotent or dominant responses (response inhibition)], working memory and its updating (addition to, or removal from, working memory of mental contents, involving content monitoring), and flexibility or shifting (switching between control rules or task demands), for each of which a host of tasks are available (105, 106) (FIGURE 4*L*). The literature also frequently employs intelligence tests (107).

#### 3.3.4. Long-term episodic memory (autobiographical memory) paradigms.

Reactions in the fear and stress reactivity paradigms described above express past experiences with stimuli or situations perceived as perceptually, conceptually, or semantically related to a test stimulus or situation. Next to the extent to which a life situation is recognized and encoded as threatening or safe (including with the help of perceptual, appetitive, and higher-order cognitive processes), the rules governing its consolidation into, and retrieval from, long-term episodic memory are therefore most likely key determinants of stress response regulation (see also sect. 3.1.4). As already alluded to in the context of extinction learning and memory, memory consolidation is a selective and active process that transforms encoded episodic content into memory traces that subserve the optimization of future behavior (108). The outcome of these processes is assessed in autobiographical memory paradigms. Here, participants are presented with neutral or emotionally valenced cues for a free recall of past-life situations, the content of which is then coded by the experimenter (109, 110) (FIGURE 4*M*). It can be postulated that remembering episodes with temporal specificity and detail rather than in a more conceptual, gistlike fashion aids threat-safety discrimination. At the same time, storing and retrieving life episodes in a conceptually generalized and abstract form, as is more frequently observed in older adults (111), is economic and necessary to be able to use past experiences in new situations (108, 111).

## 4. FINDINGS FROM BEHAVIORAL PARADIGMS

### 4.1. Detecting, Discriminating, and Remembering Threat and Safety

#### 4.1.1. Instructed and Pavlovian fear and fear extinction paradigms.

Among the most consistent findings in pathophysiological research on stress-related disorders or dysfunctions is that patients with fear- and anxiety-related disorders (including PTSD, GAD, social anxiety disorder, specific phobias, and panic disorder) relative to healthy control participants exhibit impaired CS+/CS− discrimination during differential fear conditioning (112, 113) and during delayed tests (114), in both cases characterized by heightened responding to safe stimuli (CS− or generalization stimuli) in the absence of CS+ response differences. This suggests inhibition deficits in this group of disorders. Another highly consistent finding in line with inhibitory or safety learning deficits is impaired CS+ extinction learning in fear/anxiety patients (112, 113). Less systematic evidence also links impaired extinction memory with fear/anxiety disorders (112, 115).

Observations of functional advantages of healthy control subjects in cross-sectional patient versus control comparisons may reflect pathological decline in the patients as a result of their disease. In line with the outcome-based conceptualization of resilience, identification of RFs (e.g., good discrimination or extinction ability) requires prospective-longitudinal studies. Critically, however, such studies must not only use good mental health as the to-be-predicted outcome but also control for the level of individuals’ stressor exposure. Otherwise, study participants showing better mental health outcomes than others may do so for the trivial reason that they were less exposed, not because they managed to better adapt (9, 27, 116–118). On this basis, findings that high trait anxiety, a risk factor for fear/anxiety disorders, in healthy people is associated with worse discrimination during differential conditioning and later testing (119) may suggest that individual differences in discrimination predate, or even partly determine the risk for, the development of fear/anxiety disorders; however, they cannot establish good discrimination as an RF.

Among the prospective-longitudinal studies, an early exploratory investigation found good extinction learning, but not differential conditioning, measured in male firefighter recruits (*N* = 67) before onset of their active duties, to predict lesser posttraumatic stress symptoms assessed up to 2 yr later. The analysis controlled for PTE exposure before duty onset but not during active duty, duty-related PTE exposure was unrelated to symptoms at the second measurement time point, and there was no assessment of symptoms at duty onset (120). Another similarly designed exploratory study in predominantly male fire brigade, emergency medical team, and police trainees (*N* = 99) again found an uncontrolled association between extinction (not differential conditioning) and future posttraumatic stress symptoms (121). Considerably extending these initial findings, Lommen et al. (122) reported that extinction in male soldiers (*N* = 247) before a 4-mo war zone deployment predicted symptoms 2 mo after deployment. Notably, the study controlled for deployment-related PTE exposure and predeployment symptoms, both of which had independent significant influences on postdeployment symptoms. However, no longer-term follow-up was available. Also, the effects of discrimination in conditioning were not analyzed in this study, but the very strong average differential conditioning performance (considerably stronger than extinction performance) observed in the sample suggests that differential conditioning may be too easy to acquire for healthy participants to produce sufficient interindividual variability.

To establish individual differences in threat-safety discrimination, delayed gradient-based tests are likely to be more sensitive (123), although inevitably confounded by their inherent memory component. Another sensitive discrimination testing variant may consist in first having participants learn to discriminate compound stimuli (*AX*+ vs. *BX*−) and then testing the CR to the combined presentation of the excitatory *stimulus A* and the inhibitory, or safety-signaling, *stimulus B* (124). Smaller responses to *AB* in this test in the soldiers 2 mo after deployment (subsample of *N* = 66), consistent with better inhibition, predicted fewer posttraumatic stress symptoms another 7 mo later, controlling for symptoms at 2 mo and previous PTE exposure (125). Nevertheless, one study in male and female young adults high in neuroticism (*N* = 132) reported that excitatory responses to the safe stimulus even in a simple instructed fear paradigm (where the threat-safety distinction is unambiguous by design) predict onset of anxiety disorders in the following years (126). Stressor exposure was not reported in this study.

Null results have been obtained in a similarly composed sample (*N* = 157) for differential conditioning, extinction, and extinction memory by Peng et al. (127) as well as in a conceptual replication attempt for Lommen et al. (122) by Lommen and Boddez (128) using a sample of male firefighters (*N* = 386), where differential conditioning and extinction did not predict posttraumatic stress symptoms 6 or 12 mo later. No results going in the opposite direction have been published. There are no prospective studies testing safety memory retrieval effects (in either delayed gradient-based or extinction memory tests). Of note, these null results stem from samples with relatively little exposure to PTEs. In Lommen and Boddez (128), the firefighters reported on average 5 events over 6 or 12 months, compared with 14 events over 4 mo reported by the soldiers in Lommen et al. (122) (including witnessing an explosion or being shot at in the vast majority of participants). The sample studied by Peng et al. (127) consisted of normal civilians with an average age of 20 yr.

The overall picture therefore is that good threat-safety discrimination and safety learning abilities may be RFs with protective function against the effects of eventlike stressors and against posttraumatic stress-related and perhaps other fear- or anxiety-related outcomes. The effect may be specific to these types of stressor and outcome. This preliminary conclusion is supported by the observation that the only study testing prospective associations with not only anxiety- but also depression-related symptomatology found discrimination effects on anxiety but not on depression (126). Hence, discrimination and extinction may not be general and global RFs, that is, they may not protect against the wear and tear of more chronic or hasslelike stressors and dysfunctions related to anhedonia, amotivation, despair, or exhaustion often linked with them (129).

These considerations further underline the importance of good characterization of stressor exposure in resilience studies and also suggest that resilience studies could benefit from a comprehensive characterization of stress-related impairments that permit comparison of protective effects on various psychopathological outcome dimensions.

Another general methodological consideration that is warranted by the discrimination and extinction literature concerns power. Test-retest reliabilities for typical tests used in this literature are in the poor to fair range (123), such that to detect a correlation of *R* = 0.2 (122) between a discrimination or extinction test and a psychopathological outcome with a typical reliability on the order of 0.8 to 0.9 (intraclass correlation, ICC) (130, 131) with a power of 80%, one needs >500 participants (132). For a correlation of *R* = 0.6 (120), ∼100 participants are needed. Many studies, therefore, are probably underpowered, and it can be concluded that there is a dearth of both sufficiently controlled and powered prospective-longitudinal studies on the topic.

Both methodological caveats apply, to a smaller or larger extent, to studies with most other paradigms.

#### 4.1.2. Controllability paradigms.

Although US deflation paradigms have not been made amenable to mental health research in humans yet, there is cross-sectional evidence from controllability paradigms that PTSD patients are particularly sensitive to experiencing a loss of previously learned (133) or also instructed (134) control, reacting with enhanced stressor avoidance. This effect may be more pronounced in female patients (135). Depressed patients may also be more sensitive to loss of control than healthy individuals (136). However, no prospective studies are available.

#### 4.1.3. Stress reactivity and recovery paradigms.

In patients with depression and depression comorbid with anxiety, cardiovascular reactivity to the more complex laboratory stressors typically used in stress reactivity paradigms is consistently blunted (137). These findings are in line with consistent observations that heart rate, skin conductance, startle, and facial electromyographic responses to both general and personally relevant acute stressors are blunted in patients with anxiety disorders characterized by general distress and negative affectivity [disorders of the “anxious-misery” or “general distress” dimension comprising GAD, more severe forms of (multiple trauma) PTSD, and also depression] (69, 138). Furthermore, cortisol reactivity is blunted in depression and anxiety disorders, although it is not clear yet whether this may be specific for women (139, 140). The overall blunted physiological stress reactivity in these stress-related disorders coexists with clear evidence for heightened attentional (73, 141–146) and subjective-affective (69) reactivity to disorder-relevant stimuli and for avoidant behaviors that generally characterize stress-related conditions.

Findings that blunted physiological stress reactivity scales with the number of experienced traumas and the severity of the disorder (147–149) and that patients with less disabling disorders characterized by specific fears rather than broad generalized apprehension (such as in specific phobia, circumscribed social phobia, or single-trauma PTSD) show heightened physiological responses to threat-related and negative stimuli (69, 138) suggest that blunting is part of the pathophysiological sequelae of pronounced stressor exposure and presumably an expression of stress-related exhaustion, linked with motivational deficits (150, 151).

Nevertheless, the few existing prospective-longitudinal studies indicate that good physiological reactivity to stressors is also a preexisting RF. One study in male and female participants with varying levels of mostly subclinical anxiety and depression (*N* = 1,245) observed that higher heart rate, but not blood pressure, reactivity to a paced math task negatively predicted levels of depression, but not anxiety, 5 yr later (152). The study controlled for baseline symptom levels but not for stressor exposure. A prospective relationship between higher blood pressure (but not heart rate) reactivity to a combined math, speech, and pain stressor and lower anxiety levels 3 yr later was observed in a similarly designed study in healthy adults (*N* = 102), also controlling for baseline symptom levels (153). In a study in *N* = 80 male soldiers deployed to a war zone for an average of 5 mo, both baseline symptom levels (predeployment posttraumatic stress symptoms) as well as stressor exposure (PTEs) before baseline and between baseline and follow-up (12 mo after the end of deployment) were controlled for. Exposure during deployment positively predicted symptoms at follow-up, and this effect was negatively moderated by cortisol reactivity to a speech task (154), providing the best evidence so far for a protective role of physiological stress reactivity. By contrast, cortisol reactivity to a math test and a socially evaluated pain task in *N* = 210 unmedicated healthy male and female police recruits did not predict changes in posttraumatic symptoms, negative affect, or perceived stress from before to 4 mo after a stressful 12-mo training in emergency aid services (155). The study controlled for training-related and lifetime PTE exposure and saw that training-related exposure correlated with symptom increases, although symptom increases and average final symptom levels were mild, suggesting that participants’ symptoms may be most appropriately described as belonging to the category of the less severe and less disabling dysfunctions. No findings linking reduced physiological stress reactivity with better outcomes have been reported. It is tempting to speculate that the adequate mobilization of physiological resources to challenges that appears to be a factor in the resilience against the more generalized and severe forms of stress-related impairments is driven by positive controllability or self-efficacy appraisals (sects. 2.2, 3.1.3), making effort expenditure appear worthwhile.

At the same time, not overreacting at a subjective-affective, behavioral, and attentional level also appears to be an RF, as is suggested by the literature on behavioral inhibition, a temperamental style manifesting in early childhood that is characterized by overreaction to unfamiliarity and increases the risk of developing an anxiety disorder in later life. Risk is reduced in inhibited children when they show less attentional bias to threat (less hypervigilance) and/or better cognitive control (see below) (73). In support of a protective role of normal vigilance, in a prospective-longitudinal study in *N* = 181 male and female healthy young adults, less attentional bias predicted fewer anxiety symptoms 1 yr later, controlling for baseline anxiety levels (156). There was no control for stressor exposure. In a similar sample (*N* = 70), less attentional bias moderated the effect of recent adverse life events on increases in depressive symptoms from baseline to 6–8 wk later (157). In a study in *N* = 144 male soldiers, less variability in attentional bias across trials moderated the effect of PTE exposure during subsequent war zone deployment on posttraumatic stress 12 mo after deployment (158). The study also controlled for baseline symptom levels and PTE exposure before baseline. Finally, in a study in *N* = 487 male army recruits, less attentional bias measured before training and a 6-mo war zone deployment predicted less severe posttraumatic stress symptoms 1 yr later, over and above baseline symptoms (159). The study also controlled for prerecruitment and deployment-related PTE exposure; bias moderated the effect of deployment PTEs on posttraumatic symptoms. Together these data strongly suggest that normal vigilance, as normal physiological mobilization, is protective across types of stress-related dysfunctions. A potential causal function of attentional bias in stress symptomatology is indicated by evidence for a beneficial effect of attentional bias modification training on anxiety, depression (160, 161), and anxiety reactions to real-life stressors (162).

Importantly, attentional biases in stress-related conditions are specific to disorder-congruent stimuli (e.g., to embarrassment- or panic-related stimuli in anxiety, to war- or abuse-related stimuli in PTSD, and to sadness- or discouragement-related or self-referential stimuli in depression) and extend to generally negative stimuli in the case of anxiety and depression (143, 145). Attentional bias is further increased by stimuli with personal threat relevance (163). These findings suggest that hypervigilance in stress-related disorders is at least partly driven by (rapid, automatic) learned appraisals. It nevertheless remains unclear whether the described individual differences in attention to unspecific negative information that predate stressor exposure and predict symptom development originate from individual differences in the information’s appraisal, some faster, salience-based attention allocation tendency, or a combination of both.

Stress reactivity studies often only quantify the amplitude or integral of the entire stress reaction, including its poststressor decreasing limb, without attempting to isolate the recovery phase and thus to differentiate reactivity from recovery. This probably leads to loss of valuable information, because recovery in stress tasks is more closely related than initial reactivity to affective susceptibility to real-life stressors, measured with smartphone-based ecological momentary assessment (EMA) methodology (164). Cross-sectional laboratory studies that treated reactivity and recovery separately have found that individuals high in optimism, an established psychological RF (18), have higher cardiovascular stress reactivity, whereas well-being is specifically associated with better cardiovascular recovery (165). In extension of these findings, one EMA study has linked affective recovery from real-life stressors with reduced risk status for depression (166). One single prospective-longitudinal study in *N* = 70 male and female healthy young adults investigated the rate of affective recovery from a sad mood induction and could show that faster recovery negatively moderates the effect of recent adverse life events on increases in depressive symptoms from baseline to 6–8 wk later (157). These data further suggest that, next to mobilizing resources for coping when necessary, ending resource consumption when no longer necessary may be protective, as predicted by theory (30).

Patients with anxiety disorders, including PTSD, as well as healthy individuals with high trait anxiety have a bias in favor of remembering threatening stimuli and potentially also against remembering positive stimuli when compared to healthy control subjects. These short-term episodic memory biases are especially pronounced when material is encoded in a shallow fashion (superficially) and when recall is free, that is, unconstrained and relying on explicit recollection processes (75). Memory biases in depression are observed in favor of generally negative and, to an even larger extent, against positive stimuli and are most pronounced when the encoding task is self-referential and recall is free (74). Although there are currently no prospective-longitudinal studies, these data can be taken to suggest that the normal reactivity to stressors that characterizes resilient individuals may, at least in part, be related to absence of, or reduced, memory bias for emotionally negative stimuli. As for attentional bias, it remains unclear whether negative memory bias in patients is secondary to negative appraisal biases (enhancing the encoding and/or retrieval of negative episodes), reflects some independent property of the episodic memory system, or both. One class of explicit cognitive processes determining recall rates is the inhibition (suppression) of the retrieval of unwanted memories (167), in which patients with stress-related disorders are less willing or able to engage (168). The observation that training individuals in unwanted memory suppression both induces forgetting and alleviates anxiety, depression, and posttraumatic stress symptoms (169) indicates a causal role for good suppression ability in determining level of memory bias and, eventually, resilience. The mechanistic pathway of this hypothetical effect may of course go via facilitated positive cognitive reappraisal (see below) in individuals who can more easily replace negative with neutral or positive mental contents (30, 167).

#### 4.1.4. Paradigms targeting explicit and declarative processes.

In ambiguous scenario paradigms, negative interpretive (appraisal) biases are consistently observed in social anxiety disorder and panic disorder, where they are directed toward disorder-relevant scenarios, and in generalized anxiety disorder and depression, where they are directed toward generally negative scenarios. In depression, positive scenarios are also appraised less positively (144, 170). As for attentional bias modification trainings, training attempting to modify interpretive bias is effective in reducing internalizing symptoms (171, 172).

In *N* = 84 male and female healthy young adults, less negative interpretive bias predicted less depression ∼2 yr later, over and above baseline symptoms (173). There was no control for stressor exposure. A negative predictive relationship between less bias and depression diagnosis over and above baseline symptoms, but in the absence of exposure control, was also observed in *N* = 44 currently healthy female participants, some with a past diagnosis (174). In *N* = 1,500 female healthy young adults, a lesser negative interpretive bias toward both panic- and general threat-related scenarios negatively predicted onset of panic disorder 17 mo later, again in the absence of a control for stressor exposure (175).

The experimental interpretive bias literature, though still limited in its longitudinal arm, resonates with a vast psychological literature on positive cognitive reappraisal that relies on self-report to assess the use frequency of positive cognitive reappraisal (that is, individual reappraisal tendency). This literature has shown clear associations of the construct with mental health outcomes in cross-sectional studies and also in prospective-longitudinal studies when participants are highly stressor exposed (176). An obvious expectation therefore is that this pattern extends to positive cognitive reappraisal ability (capacity, skill), as assessed with experimental tasks. Curiously, though, there is no consistent evidence that reappraisal ability is impaired in patients with stress-related disorders (177, 178) or in healthy participants low in well-being or high in stress or depressive symptoms (176). Nevertheless, good reappraisal appears to be linked with fewer depressive symptoms in the presence of additional risk factors, namely PTE exposure (179), high current stress (180), low socioeconomic status (181), or uncontrollability perceptions (182), suggesting a buffering effect of reappraisal ability.

No observational prospective-longitudinal studies have tested whether good reappraisal ability is an RF, but dedicated reappraisal trainings have achieved reduced negative emotional reactivity (183, 184) and decreased ill-being (184), further suggesting that positive cognitive reappraisal may contribute to optimal stress response regulation.

The comparatively positive general appraisal tendencies of healthy individuals, as apparent from interpretive bias tasks (as well as myriads of questionnaire studies, e.g., Refs. 82, 185, 186), have a tendency to be maintained even if there is disconfirming evidence (82), which explains their very existence in a threatening world. Specifically, beliefs about the probability of negative events are more readily updated (belief updating) when they are disconfirmed by desirable information (reduced event probability) than by undesirable information (enhanced event probability) in healthy, but not in depressed, individuals (187). This apparently healthy safety learning bias is reminiscent of, and perhaps mechanistically related to, the health value of good fear extinction learning ability (sect. 4.1.1), lesser sensitivity to loss of control (sect. 4.1.2), and positive cognitive reappraisal tendencies (above), preliminarily suggested by the reviewed literature. However, no prospective-longitudinal studies are available to date.

### 4.2. Selecting the Right Strategy

When given the choice between reappraisal and distraction in the strategy selection paradigm by Sheppes (83) and colleagues, healthy individuals who prefer reappraisal over distraction irrespective of aversive stimulus intensity score higher on mental health indicators (188), whereas data from individuals with past PTE exposure indicate that flexibly selecting strategies as a function of stimulus intensity, such that distraction is preferred at high and reappraisal at low intensity, is associated with fewer posttraumatic stress symptoms (189, 190). While the cross-sectional literature is still inconclusive, prospective-longitudinal data are lacking.

### 4.3. Supporting the Aversive System

#### 4.3.1. Perceptual discrimination (pattern separation) paradigms.

There is evidence that fear-, anxiety- and depression-related symptoms (191, 192) and disorders (193) are associated with reduced performance in the mnemonic similarity task, but there are no prospective studies.

#### 4.3.2. Reward (appetitive) paradigms.

Although globally reduced positive emotion and anhedonia (reduced motivation to obtain reward and reduced pleasure in anticipating and consuming rewards) are symptoms of PTSD and found in particular in the more severe forms of PTSD that are often comorbid with depression, it is unclear whether these reflect actual reward processing or learning deficits or whether they result from interference with reward processing during trauma reexperiencing or from avoidance of rewarding stimuli associated with trauma reminders (194, 195). The evidence for impaired reward functions is unambiguous, however, in depression (100, 196, 197). Impaired reward functions in depression are likely to at least partly underlie the interpretive (sect. 4.1.4) and short-term memory (sect. 4.1.3) biases against positive stimuli and potentially also the reduced physiological mobilization (blunting) and effort expenditure in response to stressors (sect. 4.1.4) in this disorder.

No prospective-longitudinal studies are available to date, except for one investigation in *N* = 89 male and female healthy young adults where slower attention disengagement from happy, but not neutral or disgusted, faces (an attention-based index of reward processing) prospectively predicted reductions in rumination (repetitive negative thinking) over the following 5 mo specifically in individuals exposed to a high number of adverse events during this period, which in turn predicted reductions in depressive symptom levels (198). Accordingly, training individuals to attend to positive stimuli and to positively interpret ambiguous sentences shows promise in reducing anxiety during stressful times (199).

#### 4.3.3. Cognitive control paradigms.

There is highly consistent evidence for relatively reduced cognitive performance, broadly extending across executive functions and short-term memory and inferred from tasks using nonemotional stimulus material, in pathological fear, anxiety, and depression (107). Furthermore, there is preliminary evidence for alleviation of anxiety (200) and conclusive evidence for alleviation of depression (201, 202) through cognitive control training.

Although a large body of prospective-longitudinal studies have not found a reliable predictive association of cognitive function and depression (203, 204), there is increasing evidence from a smaller and growing literature for a prospective relationship in the case of fear and anxiety. In *N* = 1,599 male and female young adults exposed to a large bushfire, better pre-PTE verbal working memory, verbal short-term memory, and attention predicted less severe posttraumatic stress symptoms 18 mo later (205). The study controlled for PTE severity, depressive symptoms, but not pre-PTE posttraumatic stress symptoms. In *N* = 668 mostly male soldiers deployed to a war zone for variable durations, predeployment short-term memory performance, but not a range of other cognitive functions, was negatively related to posttraumatic stress symptoms an average of 1.5 yr later (206). The study controlled for levels of combat intensity and predeployment symptoms, both of which had independent significant influences on postdeployment symptoms. The relationship was stronger in soldiers with higher predeployment symptoms. A 5-yr follow-up analysis confirmed these findings and also showed that immediate postdeployment memory performance was a predictor of long-term outcome (207).

A specific class of prospective studies is trauma survivor studies, where baseline assessments are performed shortly after PTE exposure (e.g., after discharge from the emergency department). Because PTE severity or acute stress symptoms at this assessment time point may impact task performance and thereby generate a false association of task performance with later symptoms that is in reality driven by these other symptom predictors, control for PTE severity and/or acute stress symptoms is particularly critical in survivor studies. In a study in 61 male and female initially healthy trauma survivors, cognitive flexibility (attentional switching) but not other functions tested 1 mo after the PTE predicted less severe symptoms 13 mo later (208). Baseline PTE exposure (trauma type) and symptoms were controlled for in this study as well as in a similar study in *N* = 138 male and female survivors, where including measures of flexibility, short-term memory, and attention obtained 1 mo after PTE in a machine learning-based prediction of PTSD diagnosis 14 mo later substantially enhanced predictive accuracy (209). Baseline symptoms were considered in a study in *N* = 101 male and female accident victims with various levels of previous exposure and current disorders and medication, reporting a negative predictive relationship of various cognitive functions 10 days after PTE with posttraumatic stress symptoms 3 and 6 mo later (210). Suggesting a causal role for cognitive performance in the protection against posttraumatic stress, an early cognitive training compared with a nonactive comparison intervention in *N* = 23 versus *N* = 26 male and female survivors enhanced cognitive flexibility and concomitantly reduced symptoms 6 mo after the PTE (208).

Beyond PTSD, a study in *N* = 2,605 male and female healthy participants found that a global measure of cognition as well as executive functions, but not short-term memory or attention, predicted generalized anxiety 9 yr later (211). The study controlled for baseline symptoms but not stressor exposure. One study in drug-using male and female adolescents (*N* = 658) did not find a relationship between executive functions and anxiety and depression 7 yr later (212).

Taken together, studies using fear- or anxiety-related outcomes, unlike studies using depression outcomes, indicate that good cognitive control, in the broad sense of the term, is an RF. The limitation to pathological fear/anxiety parallels the preliminary findings from the threat-safety discrimination literature summarized in sect. 4.1.1.

#### 4.3.4. Long-term episodic memory (autobiographical memory) paradigms.

Recall of autobiographical memories is less specific across stress-related disorders or dysfunctions (109), and meta-analysis has established that autobiographical memory specificity negatively predicts future depressive symptoms over and above current symptoms (213), suggesting that autobiographical memory specificity is an RF. However, none of the meta-analyzed prospective-longitudinal studies controlled for stressor exposure.

In the field of fear- and anxiety-related dysfunctions, a small number of available prospective studies have used a survivor design. One study in *N* = 190 male and female assault trauma survivors found that higher memory specificity 2 wk after the assault predicted less severe posttraumatic (and also depressive) symptoms 6 mo later, controlling for acute stress and depressive symptoms at baseline (214). The analysis also controlled for assault severity; however, this variable did not predict future symptoms, meaning there was no effective control for relevant stressor exposure. This study followed on two very small studies also applying control for baseline symptoms, but not stressor exposure, and reporting positive and null effects, respectively, for a prediction of posttraumatic stress symptoms by memory specificity after an accident (*N* = 22; Ref. 215) or a cancer diagnosis (*N* = 32; Ref. 216). A single study investigated memory specificity before trauma in *N* = 46 healthy male firefighter recruits (478). Here, specificity specifically in response to positive cues before the onset of active duty predicted posttraumatic stress and depression symptoms 4 yr later, over and above subclinical symptoms levels at baseline. PTE exposure at baseline, but not during duty, was controlled for.

### 4.4. Sex and Gender Effects

The prevalence of stress-related disorders is lower in men than in women (217, 218). PTSD in particular is less frequent in men, although men are more likely to experience most types of PTE (219), and this difference remains when controlling for other PTEs experienced before the index event (220, 221). This suggests that male sex or gender is an RF. It has nevertheless been discussed, especially in the context of resilience to major disasters, whether sex/gender-based risk differences may be a consequence of better access for men to support resources and less exposure of men to adversities and hassles in the aftermath of the event (222). In one study, lower rates of stress-related symptoms in male than female frontline health care workers during the first wave of the COVID-19 pandemic were no longer observed when taking into account “background” stressors such as negative effects of the pandemic on relationships or child care duties, which were more frequently reported by the women (118), although it was not clear in that study whether differences in background stressor reports resulted from veridical differences in exposure and/or a higher socially determined relevance of the reported stressors for the women (223) or were caused by a potential reporting/appraisal bias. It is also noticeable that certain forms of internalizing stress-related pathologies can be found more frequently in men, notably suicide (222), and that men generally have a higher risk for externalizing behaviors and pathologies (224), including after experiencing exposure (225). This might indicate that men are not generally less sensitive to stressor exposure than women but rather experience different stressors and/or cope with them in a qualitatively different fashion. The latter is also in congruence with the well-known differences in coping styles between the sexes/genders (e.g., Refs. 225, 226).

From the prospective-longitudinal behavioral studies on internalizing outcomes reported in this review that involved both an adequate representation of both sexes/genders and control for stressor exposure in at least some manner (that is, at least indirectly via adjustment for baseline symptomatology) (152, 153, 156, 157, 173, 198, 205, 208–211, 214–216), seven studies considered sex or gender effects but did not find any (152, 153, 173, 198, 210, 214, 227), one study found no relevant contribution of sex/gender to machine learning-based prediction (209), and two studies controlled for sex/gender but did not report their influence (205, 208). No single study demonstrated sex/gender effects. By contrast, one prospective-longitudinal study assessing internalizing outcomes during the pandemic in the general population and very carefully controlling for stressor exposure did find that male gender predicted less severe symptoms (228). This study did not employ behavioral tasks as additional predictors.

At the present moment, it thus remains open whether male gender survives as a genuine RF once exposure and objectively measured (task based) differences in behavior are factored out. The analyzed body of data is not sufficiently large to address the question of potential sex- or gender-based differences in the preference for, or ability in, employing these coping behaviors. We also note that a relevant number of studies (mainly in military personnel) were only conducted in males, such that a more systematic investigation of sex and gender influences on resilience in future studies appears one important desideratum.

### 4.5. Summary of Findings from Behavioral Paradigms

TABLE 1 gives an overview of the reviewed findings from the prospective behavioral literature, arranging the dysfunctions against which a potential RF tested in a given paradigm protects along a dimension of psychopathology from rather specific and circumscribed exaggerated fears (as in many phobias and the less severe forms of panic disorder or PTSD) to broad and generalized apprehension, despair, anhedonia, and amotivation (as in depression, GAD, or severe PTSD), sometimes termed the anxious-misery or general distress spectrum (69, 138). It is noted that the types of stressor that evoke the symptoms or generate the pathologies along this dimensional spectrum can be characterized as ranging from more circumscribed and eventlike to more chronic and/or repeated, respectively. Currently, this rough classification of stress-related mental health problems appears to best reflect the epidemiological data and also appears to provide the most suitable frame for assigning RFs to classes of stressors and stress-related symptoms. Future transdiagnostic work may generate better and more fine-grained characterization. We also note that many studies with patients reviewed in this and the following sections use classical diagnostic categories, such as depression or PTSD, and therefore make clustering of resilience-related findings on the chosen dimensional spectrum difficult. We typically consider here findings in depressed patients as relating to the generalized anxiety/depression end of the spectrum and findings in patients with pure fear- and anxiety-related disorders without comorbid depression (unfortunately often subsumed in the literature under “anxiety disorders”) as relating to the pathological fear end of the spectrum. In PTSD studies, we attempt to consider PTSD symptom severity, where possible.

**Table 1.** Findings from prospective-longitudinal studies testing the negative predictive value of behavioral paradigms for stress-related dysfunctions

Function	Behavioral Task Paradigm	Dysfunctions Negatively Predicted by the Paradigm	
		Circumscribed pathological fears (as in less severe phobias, panic disorder, PTSD)	Generalized anxiety, despair, anhedonia/amotivation (as in major depression, generalized anxiety disorder, severe PTSD)
Threat-safety discrimination, learning, and memory	Differential fear conditioning/instructed fear, fear extinction learning	+	−
	US deflation	0	0
	Controllability	0	0
	Physiological stress reactivity	0	+
	Affective and attentional stress reactivity (attentional bias)	++	++
	Affective stress recovery	0	(+)
	Episodic learning and short-term recall (valenced memory biases)	0	0
	Interpretive (appraisal) bias (ambiguous scenario task)	+	+
	Positive cognitive reappraisal ability	0	0
	Belief updating	0	0
Strategy selection	Regulatory selection	0	0
Nonaversive (auxiliary) functions	Perceptual discrimination (pattern separation)	0	0
	Reward (appetitive) processing and learning	0	0
	Cognitive control	++	− −
	Long-term episodic memory (autobiographical memory specificity)	(+)	+

Global functions considered useful for optimal stress response regulation (*left*) are tested with a variety of behavioral paradigms (*center*). Normal performances or responses in these paradigms (absence of over- or underperformance or over- or underreactions) are hypothesized to be resilience factors (RFs), that is, to show negative prediction of stress-related dysfunctions (*right*) in prospective-longitudinal studies. Dysfunctions are roughly ordered along a dimension from exaggerated fears (linked to more singular and eventlike stressors) to more generalized anxiety- and depression-type problems (linked to more chronic or repeated and severe stressors) (69, 138). Strength of RF evidence is coded as strong (++ for positive evidence, meaning confirmed presence of an effect; − − for negative evidence, meaning confirmed absence of an effect), weak (+; –), and preliminary [(+); (−)]; 0 designates absence of data. PTSD, posttraumatic stress disorder; US, unconditioned stimulus. See FIGURE 3 for overview of paradigms.

The first general conclusion that can be drawn from the behavioral literature is that there is a paucity of studies suitable to identify RFs. For many theoretically highly interesting paradigms, there are no (“0” in TABLE 1) or hardly any prospective-longitudinal studies, and many existing studies are not well controlled and are underpowered. Furthermore, there are no studies at all that are suitable to identify RPs, that is, studies where a potential RF is measured repeatedly and changes in the RF are related to changes in a mental health outcome (FIGURE 1*C*). The second conclusion is that the general idea, derived from a functional analysis of stress (see sect. 2.1), that optimized stress response regulation confers resilience finds support from paradigms that are designed to characterize aversive responding to threatening and safe stimuli, including to threat/CS+ and safe/CS− stimuli in conditioned or instructed fear paradigms and to various stressful or negatively valenced stimuli in stress reactivity paradigms. Discrimination and extinction ability in the fear paradigms appear restricted in their protective value to the more fear-related pathologies and/or eventlike stressors (they are dysfunction-specific RFs; see sect. 1.3), presumably because of the nature of the employed stimuli. But, also for protection against the generalized anxiety/depression-type pathologies typically related to chronic or repeated stressors, normal (neither exaggerated nor blunted responding) affective and attentional reactivity appears to be key, that is, normal affective-attentional stressor reactivity is a general and presumably global RF. Normal (unblunted) physiological reactivity in turn may be specific in its protective function for anxious-misery-type conditions.

Looking at the paradigms that go beyond mere stress response characterization by addressing potential underlying mechanisms of adaptive responding, the third conclusion is that absence of a negative appraisal bias is likely to be a general and global RF, which ties in well with the evidence for absence of overreactivity being broadly protective. A fourth conclusion is that good cognitive control is a dysfunction-specific RF, not extending its protective function to generalized anxiety/depression-type dysfunctions. Finally, a fifth conclusion is that autobiographical memory specificity may be an RF.

### 4.6. Spotlight on Cognitive Control and Reward System Function

Perhaps the most surprising insight is that good cognitive control does not protect against depression (203, 204), despite unequivocal evidence that cognitive control is weaker in this disorder (107). However, underperformance of depressed individuals in executive function or intelligence tests may be related to impairments in reward functions, controllability beliefs, and resulting motivation (107, 229, 230), which are cardinal feature of depression (see also sects. 4.1.2, 4.1.3, 4.3.2). This makes it possible that cognitive impairment is a secondary phenomenon and that protection against the development of depression and related conditions may be located in the preservation of good appetitive and global positive appraisal functions, a speculative conclusion that the current data do not permit us to test (TABLE 1).

If, however, cognitive control clearly protects against the more circumscribed fear- or anxiety-related dysfunctions (TABLE 1) and if it clearly helps generate more positive appraisals (by supporting positive cognitive reappraisal; Refs. 103, 104), which in turn appear to protect also against depression (TABLE 1), why does cognitive control not also protect against depression? An explanation, again speculative at this stage, may be that cognitive control is only helpful in the context of good appetitive functions. This may be because cognitive control is only employed for the purpose of downregulation of negative emotion when motivated by some positive goal, such as the prospect of a better affective state, a representation of which requires the reward system. In the absence of an appetitive goal, cognitive control may be used exclusively to support the detection of threats and the planning of threat mitigation (as in worry) or to find explanations for a current state of affairs (as in rumination), which are risk factors for the development of depression and comorbid anxiety (144). Alternatively or additionally, cognitive control may require positive mental contents to replace negative appraisals (231), which again requires good appetitive function. Taken together, the cognitive control and reward systems may interact, both being perhaps necessary but none sufficient, in preventing stress-induced dysfunctions; depression develops when the reward system is compromised. A hit to motivational functions early in the etiology of depression (and other generalized anxiety/depression-type spectrum conditions, including also severe PTSD) is also in line with the described blunted physiological reactivity to stressors that precedes it (sect. 4.1.3), which may at least partly be due to lack of appetitive motivation or perspectives (e.g., because of lack of controllability or self-efficacy beliefs).

**FIGURE 5.**
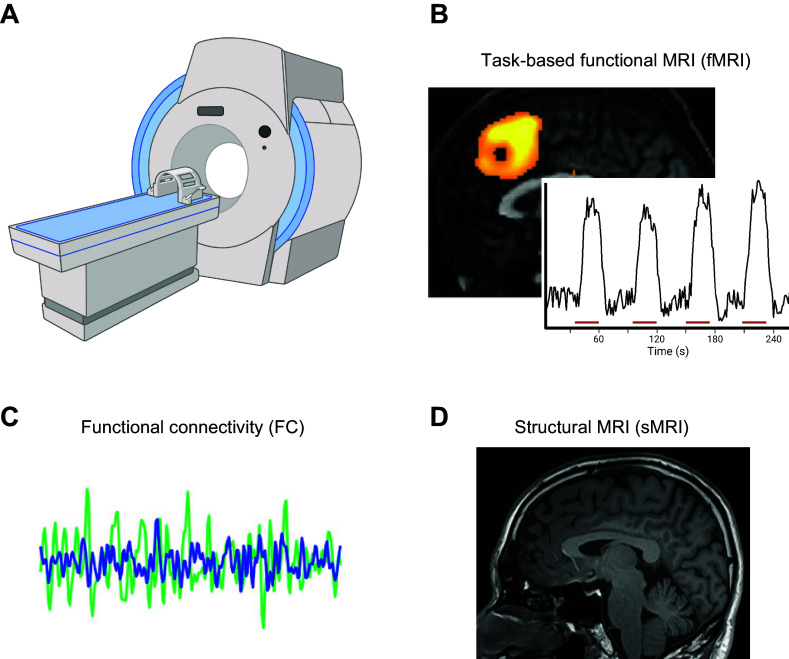
Overview of magnetic resonance imaging (MRI) methods. *A*: MRI scanner. The participant is in a lying position and may rest or receive, for instance, visual (via a mirror or goggles), auditory (via headphones), or tactile (e.g., via an electrode on the extremities) signals and may give responses via a button or keypad. *B*: task-based functional MRI (fMRI). Presentation of a task condition (red lines; e.g., a visual stimulus predicting a tactile pain stimulus) to the participant leads to neural activation in a brain area and associated increase in local blood flow and oxygenation (curve). Average signal differences between the task condition and rest (intertrial intervals) or a different task condition (e.g., a visual stimulus signaling no pain) are mapped across imaging voxels in the brain. The map shows dorsomedial prefrontal cortex (PFC) activation to threat vs. safety. *C: s*ignal cofluctuations (correlations) between 2 voxels or areas (blue and green curves) during a task (task-based functional connectivity, tbFC) or during a state of awake rest (resting-state functional connectivity, rsFC) indicate cross talk between the 2 voxels/areas or a common source of influence from another voxel/area. *D*: structural MRI (sMRI) differentiates brain tissues (e.g., gray matter, white matter, cerebrospinal fluid) to assess the size of brain areas by way of anatomical demarcation or measurement of gray matter density or cortical thickness in the area.

In addition to the brain’s systems for threat and safety processing, cognitive control, and long-term episodic memory, which can be relatively clearly linked to stress resilience (TABLE 1), these considerations justify another look specifically at the brain’s motivational system for reward. Purely behavioral analysis may miss individual differences when participants find ways to compensate for performance deficits in one system by relying on a different system. Measurement of task-related brain activity or functional connectivity (FC) with functional magnetic resonance imaging (fMRI) (FIGURE 5) can reveal such hidden differences. Furthermore, imaging researchers often apply tasks that are tailored to revealing activity differences at the expense of sensitivity for behavioral differences, making fMRI studies a unique and complementary source of information on neural RFs. Finally, task-free investigation of functional connectivity in the resting state (rsFC) and of structural connectivity using various structural MRI (sMRI) techniques (FIGURE 5) can independently reveal individual differences in neural architecture that impact the brain’s functional systems.

## 5. NEUROIMAGING FINDINGS

The analysis of the behavioral literature indicates that good threat and safety processing, cognitive control, long-term episodic memory, and likely reward processing are RFs, thereby guiding the analysis of the neuroimaging literature.

### 5.1. Methodological Considerations

#### 5.1.1. Power and reproducibility.

The same methodological caveats concerning statistical power as in behavioral studies need to be applied to neuroimaging studies, where, because of the high costs and limited availability of MRI, small sample sizes are even more common. Test-retest reliabilities for typical region of interest (ROI)-based metrics of task-related fMRI activity are similar to the reliabilities observed in behavioral fear paradigms (see sect. 4.1.1), that is, in the poor to fair range, and can be particularly low (e.g., ICCs around 0.2 or less) for the more complex tasks involving emotional or cognitive functions, as are in the focus of resilience research (232). Hence, many hundreds, or even thousands, of participants are required to obtain robust brain-phenotype correlations. The situation is better for sMRI, which has excellent reliability (232) (such that a correlation of 0.2 can be detected with ∼250 participants; Ref. 132), and intermediate for rsFC, which has good reliability (such that a correlation of 0.2 can be detected with ∼350 participants; Ref. 232). For group comparisons (e.g., PTE-exposed participants developing vs. not developing PTSD), detecting a weak difference with a *Z* score of 3.2 and a power of 80% requires from 40 to many hundreds of participants per group for a neuroimaging marker with poor reliability, from 25 to 40 for a fair marker, ∼25 for a good marker, and ∼20 for an excellent marker. The advantage of case-control studies over correlational studies is probably due to the former typically comparing extreme groups.

A specific problem of the neuroimaging literature lies in the many degrees of freedom researchers have when analyzing MRI data (233) and the richness of MRI datasets that permits researchers to compute a nearly unlimited number of tests in any single study.

To reduce the likelihood of including false positive results in this review, we therefore require, besides the application of appropriate correction for multiple comparisons in each study (http://www.humanbrainmapping.org/files/2016/COBIDASreport.pdf), that an underpowered study reporting a positive finding tests a credible and precisely defined hypothesis. A hypothesis is considered credible if it has been either published before data analysis or convincingly derived from the literature while not ignoring other obvious hypotheses that could have been tested with the dataset. A hypothesis is considered precise if it contains the tested contrast, significance threshold, and region of interest defined by a specified brain atlas or manual demarcation method. If independent replication samples are available, we alternatively also accept close replication. Underpowered studies with negative results are not considered. Studies focusing on participants suffering from traumatic brain injury as major aspect of the PTE are also not considered, to exclude physical brain damage as a source of variance.

#### 5.1.2. Study design.

As for the behavioral studies, prospective-longitudinal designs, including the special case of PTE survivor designs, are of highest interest, and control for stressor exposure and baseline dysfunctions is similarly desirable.

The PTSD literature in particular also contains cross-sectional studies that compare PTSD patients with control subjects who were exposed to PTEs comparable to the patients but did not develop PTSD (trauma-exposed healthy control subjects, TEHCs). Provided similar (matched) levels of exposure in both groups, neural differences between patients and TEHCs detected in this comparison may signify preexisting RFs and/or the operation of RPs. For instance, a smaller volume of brain region *X* in patients than in TEHCs detected at some time point after the event could indicate that TEHCs already had larger volumes in this region before the event (perhaps providing them with more buffer against PTE effects), or it could indicate that the region in TEHCs somehow better adapted to the exposure, perhaps via the recruitment of some neuroprotective molecular mechanism, in the aftermath of the event.

In these studies, the relative level of volume or function of a brain region or network in the PTE-exposed groups versus nonexposed healthy control subjects (NEHCs) is not per se a criterion to classify a THEC > patient difference as indexing a candidate RF or RP. For instance, TEHCs may also possess or develop excellent functional capacity in some stress-regulatory region that is even larger than in NEHCs. Or both patients and TEHCs may be driven by the exposure to recruit some stress-regulatory network, such that both groups functionally exceed the NEHCs, but TEHCs more than patients. Critically, however, in the absence of tight control for exposure levels, reduced volume or function in patients relative to the TEHCs may simply reflect more pronounced exposure in the patients.

### 5.2. Brain Structure

Existing large multisite studies and recent meta-analyses (234–240) comparing PTSD patients with TEHCs on brain structural indexes (compare FIGURE 5*D*) did not control for, or factor out, differences in exposure, which is typically much more severe in the patient samples. A study comparing male and female military service members and veterans with (*N* = 99) and without (*N* = 102) PTSD several or many years after combat exposure statistically controlled for the more extended exposure in the patients as well as for lifetime PTE exposure, depressive symptoms, alcohol abuse, and medication and in an additional analyses compared patients and TEHCs matched for combat and lifetime exposure (241). The study was thus able to isolate a TEHC > patient difference in left and right amygdala and left hippocampus volumes. The study did not investigate group effects in other brain areas. Another study in mainly male veterans with (*N* = 51) and without (*N* = 49) PTSD and comorbid depression and/or anxiety scanned several or many years after combat exposure, however, found relatively smaller amygdala volumes in the TEHCs, after controlling for combat exposure levels and young age at a first lifetime PTE exposure (242). Exposure itself was significantly negatively associated with amygdala volume, demonstrating the importance of taking levels of exposure into account. All participants were free from current alcohol or drug abuse, and lifetime abuse was statistically accounted for; medication was not evaluated. No other brain region was investigated. A study in male and female unmedicated survivors of the Wenchuan 2008 earthquake with (*N* = 35) and without (*N* = 36) PTSD scanned 5 yr after the event found smaller volumes in the TEHCs of the bilateral middle temporal gyri, cerebellum, and bilateral dorsolateral prefrontal cortex (PFC) (DLPFC, middle frontal gyri), corresponding to a positive relationship between PTSD symptom severity and volume in these areas, as well as a negative relationship with PTSD symptoms in the left temporal pole and the left midcingulum (243). Patients and TEHCs had similar PTE exposure levels, and these were additionally factored out in the analysis, which also controlled for depressive symptoms; alcohol or drug dependence was excluded.

An underpowered study (*N* = 13 vs. 15 mainly male unmedicated military veterans) controlled for levels of combat exposure as well as lifetime PTEs, depressive symptoms, and alcohol or drug dependence, finding TEHC > patient volumetric differences in the bilateral subgenual anterior cingulate cortex (sgACC), nucleus accumbens (NAcc), and hypothalamus, the left posterior insula, the left middle temporal gyrus, and the right DLPFC (middle frontal gyrus) (244). Another underpowered study (*N* = 14 vs. 14 male and female unmedicated police officers with matching levels of PTE exposure in childhood as well as in and outside duty, those with PTSD also exhibiting more depression and anxiety symptoms) found a TEHC > patient difference in total and left hippocampal (but not amygdalar or parahippocampal) volumes, while not investigating other structures (245). Alcohol or drug abuse was excluded. Finally, an underpowered study focusing on hippocampal subregions (*N* = 17 vs. 19 male military veterans) controlled for the number of military PTEs (though not other PTE characteristics or lifetime exposure) and found TEHC > patient volume differences in the bilateral cornu ammonis 1 (CA1)/dentate gyrus (DG) subregion as well as in the whole hippocampus (246). Alcohol and drug abuse, but not medication, were controlled for. None of the underpowered studies assessed potential differences in the time since PTE exposure.

Taken together, current results from cross-sectional patient-TEHC comparisons with adequate exposure control are disparate and partly conflicting, with the exception that all studies reporting a TEHC > patient volume difference in the hippocampus used sensitive ROI-based analysis (241, 245, 246), whereas the only well-powered study not finding the effect used less sensitive whole brain analysis (243). This suggests that the effect can be reliably detected provided appropriate methodology. The “file drawer” problem, whereby null results are less likely to be published, appears to be less of a concern in this field, because the literature contains many reports of no detectable volume differences in the hippocampus (239). Nevertheless, the conclusion that PTE-resilient individuals have larger hippocampi still requires confirmation in larger studies. Such studies should also take into consideration the question of whether a larger hippocampus volume is a hallmark of resilience specifically to PTSD-like outcomes or generalizes also to resilience to symptoms notably of the depressive (and perhaps also the substance abuse) spectra. PTSD symptom levels were moderate in the patients in the three positive studies (241, 245, 246), meaning it is unclear whether the hippocampal volume difference would also be observed with severe PTSD patients.

Meta-analyses in depression converge on evidence for larger medial PFC/cingulate cortex and insula volumes in control subjects than in patients with major depression (247–249) but like the PTSD meta-analyses suffer from not considering potential group differences in stressor exposure. Meta-analyses in fear- and anxiety-related disorders have not yet produced sufficient convergent evidence. Cross-sectional studies in the area of subclinical mood and affective dysfunctions also rarely provide an opportunity to control for influences of exposure. An exception are two very well-powered studies focusing on trait anxiety, one performed in *N* = 798 male and female young adults (students from the Duke Neurogenetics Study) who partly exhibited past or present psychiatric diagnoses. This analysis found that the significant relationship between self-reported childhood trauma and current trait anxiety levels was moderated (dampened) by the simultaneous presence of thicker orbitofrontal and ventromedial prefrontal cortices (OFC, vmPFC) and better microstructural integrity, measured by diffusion tensor imaging (DTI), of the uncinate fasciculus, a fiber tract connecting anterior temporal areas including the amygdala with OFC and vmPFC (250). Amygdala volume and integrity of the cingulum bundle, a large fiber tract connecting medial temporal, parietal, and frontal areas and also subcortical nuclei to the cingulate, did not show the effect. The other hypothesis-driven analysis was performed in an overlapping sample of *N* = 818 students from the same study and found that larger left hippocampal and rostro-dorsal mPFC volumes separately and negatively mediated the relationship between childhood trauma and trait anxiety, controlling also for recent negative life events (251). Curiously, cortical thickness and cortical volume findings in the two studies did not converge: neither OFC volume nor rostro-dorsal mPFC thickness produced the corresponding results. Potential moderating effects on symptoms of anxiety or depression have not been analyzed in the two studies. A less well-powered study in *N* = 182 mainly female adults presenting with a wide range of anxiety symptomatology did not find a moderation by hippocampus or dmPFC volume of the relationship between childhood emotional maltreatment and anxiety symptoms but instead observed a dampening influence exclusively for the volume of the right DLPFC (inferior and middle frontal gyri) (252). Hence, the current database of cross-sectional structural studies outside the field of resilience to adult PTE exposure is still too small and disparate to permit conclusions.

In the field of adult PTE research, a preregistered prospective-longitudinal study examining *N* = 210 unmedicated healthy male and female police recruits before and 4 mo after a 12-mo training in emergency aid services found that baseline volumes of the left dentate gyrus, a subregion of the hippocampus, negatively predicted increases in posttraumatic stress symptoms, negative affect, and perceived stress, while controlling for training-related and lifetime PTE exposure (253). Training-related exposure correlated with symptom increase, indicating that the sample was suited to studying PTE resilience, although increases in symptom levels and average final symptom levels were mild. Other tested hippocampal or amygdalar subregions or whole regional volumes were not associated with the outcome, which may be part of the explanation of why the cross-sectional studies only see hippocampal group differences with the more sensitive ROI-based methods. A study in *N* = 107 male and female soldiers scanned before war zone deployments that ranged from 2 to 21 mo found that larger right hippocampal volume negatively moderated the influence of deployment-related PTEs on posttraumatic stress symptoms during the deployment, while controlling for lifetime PTEs and depressive symptoms (254). At the same time, however, left hippocampal volume showed the opposite statistical effect, meaning that smaller left volume was associated with a weaker influence of PTEs on symptoms, a finding that is in apparent contradiction to the finding in the police recruits (253). Apart from sample composition and type of PTE exposure, another difference between the studies is that the soldier study was considerably less powered and also technically unable to resolve hippocampus subregions, suggesting it was globally less sensitive to detect effects while at the same time more prone to false positives than the police recruit study. These uncertainties in the interpretation of study results caused by methodological limitations further highlight the importance of power considerations. Nevertheless, viewed in combination with the earlier cross-sectional results, the best current hypothesis is that a larger volume, and presumably a better functional capacity, of the dentate gyrus is an RF that is predictive of less severe posttraumatic stress after adult PTE exposure. In the rough classification of stress-related dysfunctions in TABLE 1, dentate gyrus volume is a negative predictor of pathological fears, including simple PTSD, whereas it is unclear whether it also negatively predicts more generalized dysfunctions of the anxiety/depression spectrum, including severe PTSD.

Beyond adult PTE research, the study that cross-sectionally reported hippocampal and mPFC volume effects on the childhood trauma-to-adulthood trait anxiety relationship (251) also found that these two areas negatively moderated the relationship between future negative life events and future anxiety symptoms in a subset of *N* = 196 prospectively accompanied participants. In another prospective-longitudinal analysis of the Duke dataset (*N* = 378), it was observed that baseline uncinate fasciculus integrity moderated the relationship between future life events and future combined anxiety and depression symptoms, but only in participants reporting childhood trauma (255). These findings still stand alone, given that they cannot yet be linked to corresponding meta-analytic evidence for mood or affective disorders or symptom classes (see above).

### 5.3. Functional Connectivity

Recent meta-analyses and large multisite studies of resting-state functional connectivity (rsFC; cf. FIGURE 5*C*) in depression (247, 256–260) have yielded inconsistent and partly contradictory findings but give first hints that there may be stronger connectivity in healthy control subjects than in patients of amygdala, insula, and NAcc/ventral striatum (VS) and weaker connectivity of the thalamus with many other brain areas (247, 256, 258). Meta-analyses in fear and anxiety disorders (247, 261) and PTSD (257, 262) do not converge well. Studies assessing FC during comparable tasks (task-based FC, tbFC) are not frequent enough to permit meta-analysis.

The reward circuitry mainly comprises the ventral tegmental area (VTA), where reward-responsive dopamine neurons are located, and their major projection targets, the NAcc/VS and the ventromedial PFC (vmPFC) and adjacent rostral mPFC (263). One well-powered cross-sectional study in *N* = 926 male and female participants from the Duke cohort reported that self-reported childhood trauma and recent negative life events were positively related to tbFC in adulthood between the left VS and the rostral mPFC and adjacent perigenual ACC (pgACC) in a condition of rewarding performance feedback and that tbFC in turn was positively related to concurrent anxiety and depression symptoms, establishing a mediating relationship (264). This is a surprising finding given the strong evidence for impairing effects of childhood trauma on adult reward system integrity and function (265) and the above-cited negative association between NAcc/VS rsFC and depression (256) and would suggest that relatively poorer coupling within the reward system is an RF. Furthermore, the finding is contrasted by another, though highly underpowered, cross-sectional tbFC study in *N* = 44 male and female members of historically marginalized groups in the United States, which found that tbFC between the NAcc and the rostral mPFC and pgACC during anticipation of a reward statistically moderated (dampened) the relationship between self-reported discrimination, distress caused by the 2016 presidential election, and depressive symptoms (266). Both studies used only the NAcc/VS as seed region for connectivity testing.

In the field of exposure to PTEs in adults, one underpowered cross-sectional rsFC study in currently unmedicated male veterans, *N* = 15 with and *N* = 17 without PTSD and partial depression and substance abuse comorbidity, offered the advantage of matched combat exposure and tested group differences in coupling between an amygdala seed and the rest of the brain, finding weaker positive rsFC of the right amygdala with the right posterior insula in the TEHCs (267).

The prospective-longitudinal study in police recruits identifying larger dentate gyrus volume as a likely RF to training-induced posttraumatic stress (253) also tested a preregistered hypothesis that changes evoked by an acute stress task in the rsFC within and between two of the brain’s large-scale functional networks, namely the salience and the default mode networks (SN and DMN, respectively), would predict recruits’ resilience (155) (https://www.epanlab.nl/wp-content/uploads/2023/09/Online.Additional.Suppl-Zhang-2022-Transl.Psychiatry.pdf). The SN comprises regions like the dorsal ACC (dACC) and the anterior insula and is typically activated by salient, including emotional and stressful, stimuli (268); the DMN comprises large parts of mainly rostral mPFC and ACC, posterior cingulate, precuneus and some lateral PFC, temporal, and parietal areas and is known to be active in states of rest and in the absence of external task demands (269). Of the *N* = 190 tested participants, those in whom SN-to-DMN coupling (in particular SN-to-posterior cingulate and precuneus coupling) tended to increase from before to after acute stress showed a lesser increase in perceived stress (but not posttraumatic symptoms) from before to after training. This held when controlling for PTE exposure. The study also found that increases in coupling within the central executive network (CEN), comprising mainly prefrontal and parietal areas and known to support cognitive control and executive functions (270), predicted resilience to PTSD symptoms.

Although *N* = 190 must be considered still too small a sample size for a correlational FC study (see sect. 5.1.2), an even less-powered study scanned *N* = 98 male and female survivors of various types of PTE within 1 mo after their admission to an emergency department to predict, using machine learning, their PTSD symptoms 6 mo after admission from rsFC of the hippocampus with the rest of the brain (271). Hippocampal rsFC provided acceptable prediction. The algorithm did not predict stress, anxiety, or depression symptoms, suggesting specificity for fear-related dysfunctions (cf. TABLE 1). Medication was not controlled for.

An underpowered but strongly hypothesis-driven study in *N* = 48 male and female medical students predicted a lesser increase in anxiety levels 3 and 6 mo into a stressful medical internship from lesser coupling at the start of the internship between the locus coeruleus, the source of noradrenergic projections in the brain, and the amygdala during a task that requires the inhibition of emotional information (272). The analysis controlled for past PTE exposure, anxiety levels at baseline, and the number and severity of adverse events experienced during the internship.

Note that all these longitudinal studies are recent and tested different specific hypotheses, which is why no information about replicability or generalizability of findings is available. It can, however, be assumed that older, very prominently published reports prospectively relating positive hippocampus-to-vmPFC coupling (273) and negative amygdala-to-midcingulate coupling (274, 275) during presentation of negative emotional picture material to resilience in underpowered samples of military personnel have not been replicated, given the absence of corresponding publications.

Overall, the functional connectivity literature is still too disparate to draw conclusions about general RFs, or also patterns of RF specific to certain types of stressor or stress-related dysfunction. Cautiously, the finding that connectivity of the hippocampus with the rest of the brain permits prediction of PTSD (271) may be combined with the reviewed structural MRI findings to postulate a role for proper hippocampal function in resilience to PTEs. The meta-analytical finding of better VS/NAcc connectivity in control subjects relative to depressed patients (256) in combination with the two cross-sectional studies finding variation in reward system connectivity as a function of resilience to anxious and/or depressive symptoms (264, 266) point toward a possible involvement of reward system functioning in resilience against these types of impairments. However, the cross-sectional nature of these data means that they do not extend the existing behavioral literature, which is also restricted to cross-sectional studies (see sect. 4.3.2), and that such a conclusion based only on the functional connectivity literature would therefore be premature.

### 5.4. Brain Activation

Meta-analyses have begun to find first evidence for less activation (cf. FIGURE 5*B*) in the left amygdala and hippocampus in healthy control subjects relative to patients with anxiety disorders, depression, and PTSD in a variety of emotional and cognitive tasks and independent of medication status (Ref. 276, but see Ref. 277 for subthreshold and Ref. 278 for null findings). Meta-analyses focusing on reward processing in depression have shown stronger activation in ventral striatum in control subjects (279, 280) and, interestingly, also a prospective association between stronger ventral striatum activation and less severe depressive symptoms (280), thus providing initial longitudinal evidence for a potential protective role of good reward system function that is not available so far from the purely behavioral task literature (see sect. 4.3.2). One meta-analysis focusing on cognitive control tasks reported stronger activation in control subjects than in medication-free patients from a large variety of nonpsychotic mental disorders in the dACC/dmPFC (281). None of these analyses considered the influence of stressor exposure.

Whereas meta-analyses based on many small-sample studies encounter their own problems (282), the evaluation of single brain activation studies reveals considerable difficulties in finding consistent evidence for neurobiological RFs, difficulties that are even more pronounced than in MRI studies of brain structure and functional connectivity, most likely due to the much poorer reliability of task-based fMRI metrics (232). This problem is illustrated by a recent failure to replicate findings from a large cohort of *N* = 146 PTE survivors that PTSD symptom development can be predicted based on activation profiles in fMRI tasks using aversive facial and rewarding monetary stimuli (283) in an independent similar cohort of *N* = 130 survivors (284). In the same vein, the finding that reactivity of the amygdala to aversive stimuli in the same face processing task as used in the PTE survivor studies prospectively moderated (dampened) the relationship between negative life events and anxious and depressive symptoms in *N* = 340 young healthy adults (285) was not replicated in two later analyses of a partly overlapping sample [*N* = 196, stratified for presence or absence of childhood maltreatment (286) and *N* = 120 (287), all from the Duke cohort]. Also, in an independent sample of *N* = 156 young men from low-income backgrounds (and thus presumably with higher than average stressor exposure), amygdala reactivity to aversive faces did not predict future depressive symptoms (288). Although nonreplication in smaller samples does not necessarily imply that the original finding is not true, it highlights that the field must rely on considerably larger samples than currently available to most researchers. One study in *N* = 804 male and female young adults (students), partly presenting with psychiatric conditions, finding that activation of the ventral striatum to rewarding positive feedback moderated the effect of self-reported childhood trauma on anhedonia symptoms inspires hope (289) but, because of its cross-sectional nature, only allows limited conclusions. In synopsis with the above prospective meta-analysis (280), the limited conclusion is that good reward system function is a candidate neurobiological RF in the context of depression.

### 5.5. Summary of Neuroimaging Findings

The most valuable insight from the neuroimaging literature on resilience is the moderate evidence for good hippocampal, and in particular dentate gyrus, structure and function being an RF against the development of posttraumatic stress symptomatology, namely the less severe forms of the pathology, after PTE exposure in adulthood.

Another valuable insight from the neuroimaging literature is the preliminary evidence for a role of the reward system as an RF against depression-type symptomatology. Although the behavioral task literature already indirectly suggested this conclusion (see the discussion in sect. 4.6), the neuroimaging literature additionally provides data also of a prospective relationship between good reward function and decreased risk for depression, albeit in the absence of stressor exposure control. Notably, this insight relies on neural, not behavioral, task-based metrics, demonstrating the added value of functional brain imaging. By contrast, the literature on structural interindividual differences provides few hints on the reward system, which might indicate that dysfunctions with etiopathological relevance for depression may not reach a level that manifests in macroanatomical abnormalities, unless perhaps where such dysfunctions have a basis in early-life adversity and their consequences manifest in a generalized anxiety/depression-type symptomatology (250).

There is considerably less evidence for normal amygdala reactivity to threat-related stimuli as an RF, the evidence being limited to a single brain activation study on negative life events and associated anxiety and depression symptoms in a relatively mildly exposed population (285). Nevertheless, normal amygdala reactivity remains a viable hypothesis. Evidence for a role of cognitive control systems, that is, essentially the dorsomedial and lateral PFC (290), is largely lacking (except Ref. 155), but the data also do not exclude this possibility (see, for instance Ref. 291).

Globally speaking, the neuroimaging literature is marked by a dearth of sufficiently powered and well-controlled prospective-longitudinal studies. This means that the space of potential macroanatomically localizable RMs is still vastly underexplored and many discoveries may still lie ahead. Nevertheless, the current state of empirical human resilience research, both on the level of behavioral task and on the level of neuroimaging, provides enough hints that require theoretical integration.

### 5.6. Integrating the Behavioral Task and Neuroimaging Literatures

An important starting point for the endeavor of integrating the behavioral and neuroimaging literatures must be the documented role of the dentate gyrus in pattern separation. Pattern separation refers to the storage of event memories as distinguishable from those associated with other, but similar, events or situations and their disambiguation at later confrontation with such events (292). Pattern separation also underlies the ability to recognize a threat-associated event as safe when its occurrence in a context different from the context in which it originally occurred signals absence of threat, and as such also permits extinction of context-driven fear memories (292). The dentate gyrus is therefore probably a key neural substrate for threat-safety discrimination and safety learning and may also support fast recovery after threat termination, positive appraisal of ambiguous scenarios, and autobiographical memory specificity, all functions that protect against the exaggerated and overgeneralized fear reactions that are characteristic of the fear-related disorders, including the simpler forms of PTSD (see *left* dysfunction column in TABLE 1). Cognitive control functions can provide an alternative or complementary route toward differentiated appraisal and optimal stress response regulation (sect. 3.3.3), which is likely to be the reason why they figure prominently as RFs against pathological fears (see also TABLE 1).

Dysfunctions of the generalized anxiety/depression spectrum (*right* dysfunction column in TABLE 1) are usually tied less to single specific event memories or concrete objects of fear but more to chronic stressor exposure or adverse circumstances and associated categorical beliefs of pervasive danger and hopelessness. These may in many cases reflect a realistic appraisal, and discrimination may be less helpful in such circumstances, simply because an objective analysis of the threatening aspects of a situation may not identify the islands of safety where one can relax. By contrast, more positive appraisals may be obtained from a simultaneous recognition of the rewarding aspects of a situation or its associated opportunities and the construction of more balanced world- and self-models that do not exclusively focus on negative information. Thus, by seeing the bright side of things too, notions of doom (world model) and helplessness, worthlessness, or guilt (self model) may be prevented or outweighed. In the same way that threat-safety discrimination necessarily requires the dentate gyrus, “seeing the bright side” necessarily requires the reward system. Activation of the reward system further has the welcome consequence that it antagonistically inhibits activation of the aversive system (92–95). Through these routes, longer-term excessive stress responses, which can eventually lead to hyporesponsiveness (blunting) of the stress axes (293, 294) as an early sign of developing depressive pathology (see sect. 4.1.3), become less likely under chronic-circumstantial and/or extreme adversity. Good cognitive control capacity, which is clearly not an RF in the context of generalized anxiety and depression (TABLE 1), is not helpful against these types of dysfunction precisely because individuals with a reward system dysfunction fail in the first place to generate the positive mental contents that cognitive control could prioritize in working memory. They also fail to motivate themselves to pursue possible rewards and thereby to reinforce potential positive appraisals.

**FIGURE 6.**
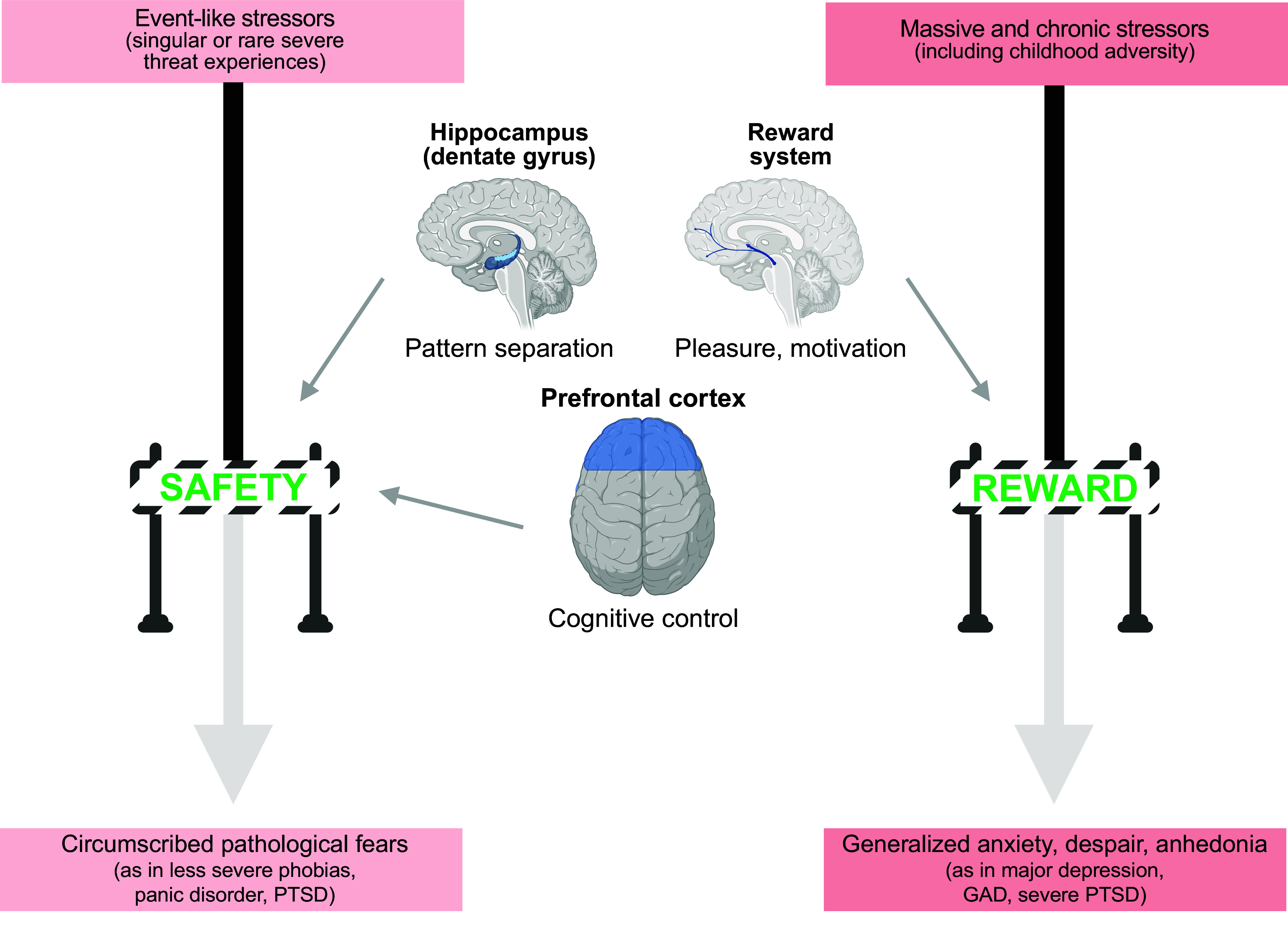
Working model for the functional neuroanatomy of resilience. Hippocampus (dentate gyrus)-based pattern separation and prefrontal cortex (PFC)-based cognitive control help recognize and exploit safety during or after exposure to singular and eventlike stressors [e.g., potential traumatic events (PTEs)]. This permits threat-safety discrimination and safety learning, overall more positive appraisal, and, eventually, generation of optimized (nongeneralized and quickly recovering) stress responses (sect. 2.2; FIGURE 2 and FIGURE 3). Development of circumscribed pathological fears [such as in the less severe phobias, panic disorders, or forms of posttraumatic stress disorder (PTSD)] becomes less likely. The reward system helps recognize and exploit rewards existing within contexts of more massive or chronic stressor exposure and in their aftermath. This also permits overall more positive appraisals and optimal stress responding and protects against the more severe and generalized dysfunctions of the generalized anxiety/depression spectrum [including depression, generalized anxiety disorder (GAD), and severe PTSD] often following these stressors (sect. 4.5; TABLE 1).

These conclusions from the existing empirical findings on a background of stress regulation and appraisal theory give rise to a neurobiological working model of resilience centered on the hippocampus, the PFC, and the reward system, whereby hippocampal (dentate gyrus)-based pattern separation and PFC-based cognitive control protect against the development of circumscribed pathological fears, whereas reward system-based pursuit and savoring of positive reinforcers protect against the development of generalized anxiety and depression and more severe forms of PTSD. Both mechanisms contribute to an overall more positive appraisal of, and relatively milder stress reactions toward, either eventlike episodic or chronic-circumstantial stressors and, when maintained over longer times, reduce the allostatic load and associated disease probability coming with these stressors (FIGURE 6). A lesser propensity for stress may be reflected in lesser amygdala and stress axis reactivity to aversive stimuli.

The idea of good integrity and function of hippocampus, PFC, and reward system in times of stress being key for resilience by benefiting positive appraisal effectively provides a neuroanatomical-functional implementation of positive appraisal style theory [positive appraisal style theory of resilience (PASTOR); Ref. 30], introduced above (sect. 2.2, FIGURE 3*B*). At this stage, this working model is open to extension and refinement, e.g., by future evidence for mediation of hippocampus, PFC, or reward system influences through amygdala reactivity or for independent (add on) protective roles of better resistance of the amygdala or of the stress axes to the longer-term overstimulation that becomes more likely when hippocampus, PFC, or the reward system fails. In the classification system of RFs laid out in sect. 1.3, hippocampus (dentate gyrus), PFC, and reward system functions all are stressor- as well as dysfunction-specific RFs (nonglobal and nongeneral RFs). The resulting broad positive appraisal tendencies are a general RF. The neurobiological explanation afforded by this working model for positive appraisal tendencies does not exclude other, namely sociocultural, influences on appraisal style. There is no obvious indication in the reviewed human data leading to this proposal that these RFs are sex or gender specific.

The hippocampus-PFC-reward system working model will provide a red thread for the analysis of other biological findings in resilience.

## 6. NEUROBIOLOGICAL RESILIENCE MECHANISMS

A neurobiological framework of resilience focusing on the hippocampus, the PFC, and the reward system (the VTA, the VS, the vmPFC) cannot avoid starting with two preclinical observations. First, the hippocampus is the brain structure most vulnerable in its integrity and function to longer-term increased concentrations of circulating glucocorticoids (in humans mainly cortisol, in rodents corticosterone; in the following: CORT), found in situations of repeated strong stress reactions associated with HPA axis activation, whereas the PFC and ventral striatal regions are among the second most vulnerable target regions of CORT (84). Second, the hippocampus, the PFC, and the VS are the areas of the brain where stress has its strongest effect on the integrity of the blood-brain barrier (BBB) (295, 296), the endothelial interface that controls most of the bidirectional humoral communication between the central nervous system and the circulation. Chronic stress is associated with a low-grade systemic inflammatory state (293) that can in turn lead to disrupted function of the BBB (297–299). Depending on the chosen pathogenetic model, these BBB disturbances allow the entry of potentially neurotoxic proinflammatory factors such as the peripheral interleukin IL-6, or also monocytes, into the brain parenchyma (300, 301) or they restrict clearance from the brain parenchyma of excess glutamate and depress brain energy metabolism (299).

Hence, it occurs that the brain areas for which the evidence that they matter in resilience is strongest are also the areas that are most vulnerable to the two major routes by which pronounced stress exerts detrimental molecular effects on the brain. This immediately leads to the hypothesis that resilient individuals stay healthy despite stressor exposure because their hippocampi, prefrontal cortices, and reward systems are particularly resistant through some intrinsic properties or extrinsic factors to stress-associated functional impairment. It turns out that, in reviewing the literature on molecular, cellular, or circuit-based RMs in the brain, we predominantly find evidence for such mechanisms in these three regions or systems.

### 6.1. Animal Models of Resilience

Animal models have two key advantages over human experimental approaches: the level of stressor exposure can be more easily controlled, and invasive measurements and manipulations can be more easily performed.

Outcome-focused models recapitulate the definition of human resilience as maintained mental health despite adversity (sect. 1.1, FIGURE 1*A*) by subjecting the experimental animal to a defined stressor and capitalizing on the considerable heterogeneity in behavioral outcomes that can be observed in subsequent tests even in genetically identical animals of the same inbred strain (e.g., C57BL/6 mice). This outcome heterogeneity allows stratification of animals into defined subgroups, that is, “resilient” animals that maintain normal behavior comparable to a nonstressed control group and “non-resilient” or susceptible animals that are severely affected by the exposure.

**FIGURE 7.**
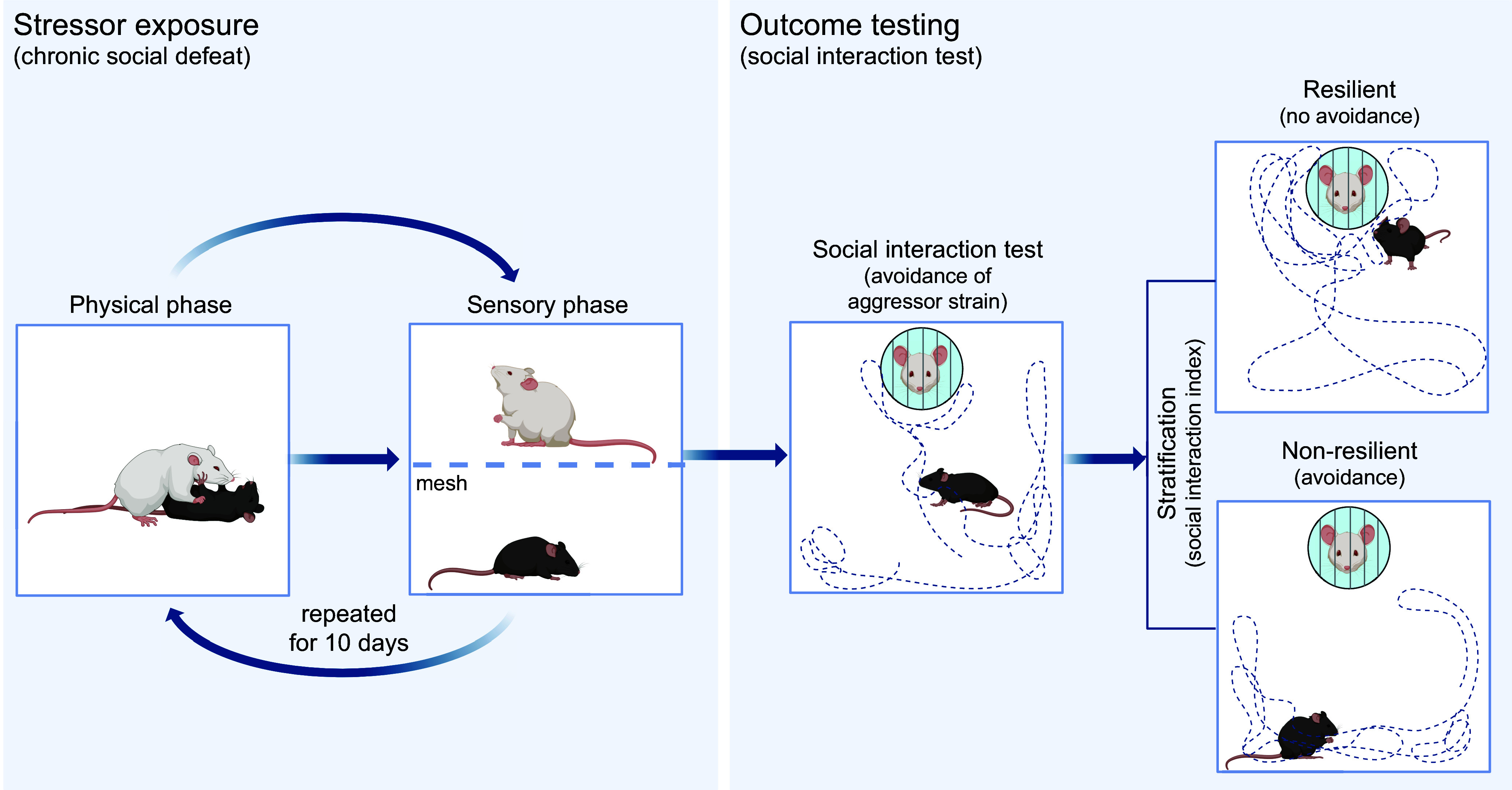
Chronic social defeat (CSD) and social interaction test. In CSD, the test mouse (intruder, brown) is placed in the home cage of an older and larger aggressive resident mouse (white). After a short phase of physical contact involving aggressive encounters, a mesh separates the 2 mice for the next 24 h (sensory phase), until the procedure is repeated in the home cage of another resident. After a chosen number of days (e.g., 10) and a chosen interval after the last defeat session, a social interaction test is performed to quantify how much time the test mouse spends exploring and interacting with an unfamiliar mouse from the resident’s strain, placed below a mesh. Mice that avoid interaction with the resident strain are considered non-resilient (*bottom*), whereas animals that show social interaction times comparable to nondefeated control animals are considered resilient (*top*).

One particularly successful and popular stressor used in outcome-focused approaches in mice is the chronic social defeat (CSD) paradigm (FIGURE 7), where a male intruder (test mouse) is placed in the home cage of an unfamiliar male resident, typically a retired breeder that is older and larger than the intruder and has been preselected for aggressive behavior (302, 303). After a defined period (e.g., several minutes) of frequent aggressive behavior by the resident toward the intruder, both mice are kept in continuous sensory contact behind a clear perforated barrier for 24 h. The same dyadic defeat element followed by continuous sensory contact is repeated daily (for 10 consecutive days in most studies, each time with a new resident). Several adaptations of the paradigm for female mice have more recently been developed (304–307), but their ecological validity is still debated (308).

The most popular outcome test after CSD is the social interaction test, which builds on the innate drive of mice to explore, and socially interact with, conspecifics. When given the opportunity to freely explore a mouse from the resident’s strain that is placed below a mesh to prevent potential aggressive attacks in this test, a considerable number of test mice shows markedly reduced social interaction relative to nonstressed control animals, a phenotype considered to reflect impairments in motivated social behaviors as frequently observed in depression or other mood and affective disorders (309). Maintained interaction levels are interpreted as resilience to CSD-induced social avoidance. The introduction of this relatively simple, user-friendly, and high-throughput behavioral assay has inspired a large number of neurobiological investigations. Other, less frequently employed outcome tests assess potential anhedonic and amotivational effects (e.g., via sucrose preference, forced swim, or tail suspension tests) and anxiogenic effects (via elevated plus-maze or open field tests) of the exposure.

Although CSD is a chronic psycho-social stressor, other models employ nonsocial (physical) chronic stressors, such as repeated restraint in a narrow tube (310), or also more eventlike physical stressors, such as unsignaled strong electric foot shocks or fear conditioning, the latter sometimes exacerbated through additional concurrent stressors (311). Outcome classification following nonchronic paradigms also often uses fear extinction or acoustic startle reactivity (311) and may thus capture phenotypes like impaired safety learning or hyperarousal, reminiscent of PTSD.

In all outcome-focused paradigms, neurobiological differences between non-resilient animals on one hand and both resilient and control animals on the other hand are informative about potential pathogenetic mechanisms, whereas the critical comparison from a resilience perspective is between the resilient animals on one hand and both the non-resilient and control animals on the other hand. This means that, although findings specifically in the non-resilient group may be highly relevant for identifying predispositions or processes involved in disease, they contribute relatively little to our understanding of RMs and are therefore not covered in this article.

**FIGURE 8.**
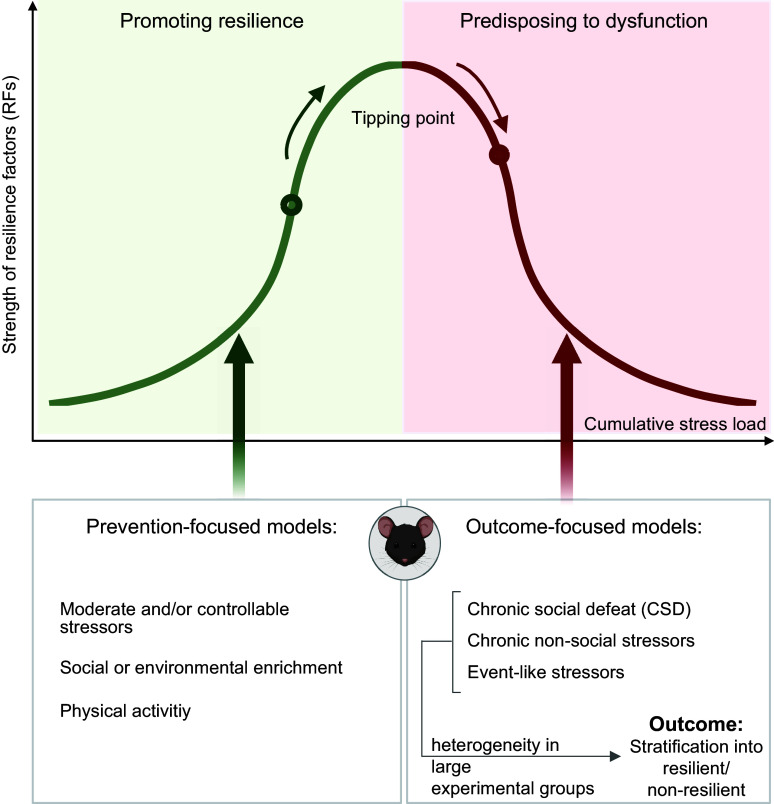
Distinction between outcome- and prevention-focused models. In prevention-focused approaches, resilience factors (RFs) are believed to be strengthened through exposure to positive or moderately activating and challenging conditions (*top left*). This results in reduced reactivity to stressors (better stress response regulation, see sect. 2.1) later in life. In outcome-focused approaches, conditions are intended to be so stressful that RFs are impaired and/or pathogenetic processes are initiated in most animals (*top right*) and normal adaptive behavior is impaired. An underlying assumption is that there will be a tipping point beyond which cumulative allostatic effects of stressor exposure damage the system. Resilience-promoting interventions in prevention-focused models must remain within a range that does not push the individual over the tipping point.

The outcome-focused models can be differentiated from approaches that are designed to actively promote resilience. These prevention-focused models provide animals with positive or moderately challenging and activating experiences, often placed early in life, including physical activity, being reared in communal nesting or an enriched environment, or escapable (controllable) foot shock (see sect. 3.1.2, FIGURE 4*B*) with the intention to reduce the animals’ aversive responses to severe stressors later in life. Such effects have indeed been reported (e.g., Refs. 64, 312–320). Insofar as these experiences can be classified as stressors, the manipulation emulates the human phenomenon of stress inoculation (sect. 1.1 and FIGURE 1*D*) and can thus be informative about a potential positive side of adversity, consisting in the strengthening of RFs (the induction of RPs) (FIGURE 8).

### 6.2. Hippocampus (Dentate Gyrus)

Latest data indicate an almost exclusive capacity of the dentate gyrus in generating new neurons (granule cells) postnatally (321, 322). Adult hippocampal neurogenesis, through integration of newborn neurons into existing neuronal networks and their impact on network activity, plays a major role in hippocampus-dependent flexible control of behavior (323). Newborn neurons are believed to channel incoming excitation into nonoverlapping subnetworks, leading to pattern separation and flexible integration of novel information into familiar contexts (324).

In a landmark study, selective silencing of adult-born granule cells in the ventral dentate gyrus, using an in vivo chemogenetic approach during a short (5 days) social defeat protocol that normally does not induce social interaction deficits, led to a significant reduction in social interaction and also increased anxiety-related behavior immediately after the last day of defeat, indicating substantially heightened aversive processing of a relatively mild social stressor. This was associated with increased defeat-induced activity and in vitro excitability of the mature (old) granule cells in this region (325). These findings were supported by complementary gain-of-function experiments in which neurogenesis was boosted in the weeks before a standard (10 day long) CSD, leading to a reduction of CSD-induced social avoidance, anxiety, and activity and excitability of mature granule cells immediately after defeat. Subsequently, a protective effect of enhanced neurogenesis against the detrimental effects of a later 4-wk unpredictable chronic mild stress paradigm on active coping motivation (struggling in the tail suspension test) and cognitive performance (water maze task) was shown (326).

Importantly, adult-born granule cells can inhibit mature granule cells (327), suggesting that adult hippocampal neurogenesis may have contributed to biasing information processing away from negative content in these experiments. Besides negative experiences, positive experiences can also leave memory engrams in the dentate gyrus, whose artificial (optogenetic) reactivation during behavioral testing after chronic immobilization stress reduces the amotivational and anhedonic, but not the anxiogenic, effects of the stressor, by enhancing activity in an excitatory dentate gyrus-basolateral amygdala-NAcc pathway and concomitant glutamate and dopamine release in the NAcc (328). Longer-term (5 days) optogenetic activation of positive engrams in the dorsal dentate gyrus after immobilization had similar behavioral effects and also reversed the immobilization-induced attenuation of hippocampal neurogenesis, an effect that correlated with its antianhedonic effects. Hence, the hippocampus can alleviate stress by channeling activation during or after stressor exposure to the reward system (see below), on the basis that past positive experiences are remembered, and this effect may be supported by, or lead to, beneficial long-term plasticity in the dentate gyrus, in the form of neurogenesis.

Neuroplasticity is a broader concept that includes lasting changes at several levels, besides neurogenesis including changes in spine and dendritic morphology (structural plasticity), in synaptic function (functional synaptic plasticity), and in the molecular and cellular mechanisms that accompany such changes (329). Further supporting a role for hippocampal plasticity in resilience, it was shown that the described broad preventive (proresilience) effects of prestressor systemic ketamine treatment (330) are at least partly mediated by long-term induction specifically in stressed mice of the immediate-early gene and transcription factor ΔFosB in the dentate gyrus output region, CA3, of the ventral hippocampus (331). Hence, ketamine modifies the stressor experience, in line also with findings that it changes neural activity during encoding of contextual fear memories in ventral CA3 (331), and this improves adaptive behavior in different subsequent tests in a plasticity-dependent manner. Interestingly, ketamine pretreatment does not alter conditioned fear responses but facilitates discrimination (sect. 3.1) between threatening and safe contexts (331), suggesting a direct effect on hippocampal pattern separation and, perhaps, the formation of positive (safety) engrams.

A final piece of evidence for hippocampal plasticity effects on resilience comes from the observation that overexpression of the gene for the neurotrophin brain-derived neurotrophic factor (BDNF) in the dorsal dentate gyrus before unpredictable chronic mild stress abolished stress effects on later hedonic, exploratory, and active coping behaviors (332).

Importantly, the reviewed experiments do not address whether any of the described mechanisms (neurogenesis, positive engram retrieval, ΔFosB expression, BDNF expression) occurs naturally in resilient individuals in the context of stressor exposure and there exerts protective actions, such that it can be classified as an RM (FIGURE 2*A*) or as a basis for an RP that lastingly changes system function in an adaptive way (FIGURE 1*C*). A particularly valuable finding is therefore that CSD itself induces ΔFosB expression in glutamatergic projection neurons from ventral hippocampus to medium spiny neurons in the NAcc and that this is critical for maintenance of social interaction in a later test (333). CSD-induced ΔFosB expression is associated with reduced excitability in this pathway, a phenotype that has previously been observed to characterize CSD-resilient mice (334). The pathway is different from the pathway via the basolateral amygdala, whose excitation carries the beneficial effects of positive engram reactivation (328). This further underlines the notion that hippocampus-dependent RMs involve a shift in the balance between aversive and appetitive memories. It also highlights that the NAcc activation as such is not a sign of resilience and different cell populations and circuits must be considered separately, as we discuss in sect. 6.4 on the reward system.

In addition to prevention with ketamine, prevention-focused approaches using communal nesting, environmental enrichment, or physical exercise (FIGURE 8) also promote hippocampal plasticity, as suggested by reports of increased hippocampal BDNF levels (335), increased number and survival of newly generated neurons (319, 335–338), and better functioning of adult-born neurons (319, 339). Indirectly, these data strengthen the link between hippocampal plasticity, pattern separation, and resilience.

Although the rodent hippocampus literature thus supports its protective role, in agreement with the conclusions from the human data (sect. 5.6, FIGURE 6), it does not indicate that hippocampal RMs protect only against the development of circumscribed, pathological fears. Rather, it appears that the hippocampus may also contribute to resilience against dysfunctions that can better be characterized as mimicking human depression, presumably via its links with the reward system.

### 6.3. Prefrontal Cortex

The functional-neuroanatomical working model of resilience inspired from the human data (sect. 5.6, FIGURE 6) postulates PFC-mediated cognitive control as a complementary pathway toward detecting and exploiting safety. Using the controllability paradigm (sect. 3.1.2, FIGURE 4*B*), Maier and Seligman (64) have established that male rodents exposed to escapable, that is, controllable, shocks activate an inhibitory connection from the medial PFC (mPFC) to serotonergic cells in the dorsal raphe nucleus in the brain stem, which are responsible for mediating the stress response to the shocks through projections to the amygdala, the periaqueductal gray, and the striatum. The controllability experience further induces protein synthesis (340)- and NMDA-type glutamate receptor (341)-dependent plasticity in this circuitry, such that mPFC-based control over the dorsal raphe is also present when the animals are later exposed to other stressors, including stressors that are uncontrollable, presented in different contexts, and presented after an extended time. This goes along with reduced stress responses. That is, the controllability experience inoculates animals (FIGURE 1*D*) against an array of stressors by inducing a neural RP (lastingly enhanced medial prefrontal control over the serotonergic dorsal raphe).

From the functional-mechanistic perspective on stress response regulation (sect. 2), it obtains that dorsal raphe nucleus inhibition by the mPFC instantiates the positive appraisal of stressors on the controllability dimension, one of the three key dimensions of stressor appraisal (sect. 2.2), and the associated reduced acute stress reactivity. This makes it a prime candidate for an RM. Importantly, the observation that the beneficial effect of controllability manipulations on stress responses persists into many different, including objectively uncontrollable, stressor situations means that the induced controllability expectations do not reflect mere instrumental learning of concrete action-outcome contingencies (64). They rather appear to express a more abstract perception of general manageability or mastery of stressful situations that is immune to stimulus and context changes and to single experiences of uncontrollability. Immunity to control disconfirmation is an apparent parallel to the observation that healthy humans, but not patients with PTSD or depression, are relatively insensitive to loss of control (sect. 4.1.2). More generally, this also parallels the reported tendency of healthy people not to update beliefs about negative outcome probabilities when they are confronted with information that they have underestimated a probability (sect. 4.1.4). Hence, resilience in animals, too, appears to be related to some relatively stable level of threat underestimation, as postulated by positive appraisal style theory (sect. 2.2). Immunity to stimulus and context changes, on the other hand, underlines the generalized protective nature of the appraisal tendency established by the controllability experience, suggesting that this positive control appraisal bias is a global RF, that is, it protects against the effects of many different stressors and is therefore a particularly interesting target for resilience-promoting interventions (30, 312) (sect. 1.3). This is reminiscent of the unexpectedly global protective function of hippocampal RMs, discussed above.

Female rodents show weaker short- and long-term controllability effects than male rodents, because of more prolonged increases in prefrontal extracellular dopamine levels during the stressor, which shifts behavior in the controllable condition toward habitlike responding and may thus impede the detection of controllability resulting from making a goal-directed successful control effort (342). Similar mechanisms may underlie the higher sensitivity of female PTSD patients to loss of control (see sect. 4.1.2).

Further support for a role of plasticity in the PFC in stress inoculation comes from a study in monkeys that provided animals with a moderate (intermittent) and hence presumably controllable form of stressor experience in childhood, which led to reduced stressor responsivity in adulthood relative to a control group not exposed to the childhood stressor. The effect could be statistically explained by enhanced mPFC-to-subcortical functional connectivity in adulthood in the inoculated animals (320). The same procedure also improves later prefrontal-dependent control in a nonemotional response inhibition task (343), suggesting a general boosting effect of stress inoculation on control-related PFC functions.

These animal experiments confirm the notion that good prefrontal function is critical for resilience (sect. 5.6, FIGURE 6) and indicate that stress inoculation and/or experiences of mastery and control may strengthen this presumably global RF, in the sense of a prevention approach (FIGURE 8). As in the hippocampus, learning and memory functions supported by neural plasticity are crucial.

### 6.4. Reward System

The human data indicate that the reward system may protect against stress-related dysfunctions of the more severe form, such as found in depression, comorbid anxiety, or multiple-trauma PTSD, by facilitating the detection and exploitation of rewards, or positive experiences beyond safety (sect. 5.6, FIGURE 6). Induction of resilience by reactivation of past positive experiences in the hippocampus, leading to glutamate and dopamine release in the NAcc (sect. 6.2), supports this idea.

During defeat in the CSD paradigm, male mice that will later be classified as resilient by way of the social interaction test spend more time fighting back at the aggressive resident and taking a vigilant posture where they face the aggressor than non-resilient mice. Mice of female sex generally hardly fight the aggressor but, when resilient, also vigilantly face it (344). Notably, resilient and non-resilient mice experience a comparable amount of attacks, suggesting that the critical difference between them lies not the differences in objective exposure to the stressor, but in the way in which they perceive or respond to the stressor. This is in agreement with the general notion that stress response regulation is a central determinant of resilience (sect. 2.1, FIGURE 2*A*) and is further supported by recent investigations of the reward system during these behaviors.

Investigation of the reward system in the context of resilience to stressor exposure (e.g., Refs. 345, 346) has been complicated by the fact that dopamine release in the NAcc is induced not only by unexpected rewards but also by stressors (347) and that the VTA contains different populations of dopaminergic NAcc projection neurons in different VTA and NAcc locations, some responsive to rewarding, some to aversive stimuli, or a combination (348, 349). Furthermore, NAcc-projecting dopamine neurons may also release BDNF in the NAcc, which unlike in the hippocampus has been shown to promote depression-like effects of CSD (e.g., Ref. 350). Finally, downstream effects of dopamine release in NAcc are mediated by two types of GABAergic medium spiny neurons (MSNs) with different functional properties (351).

Significant progress in the elucidation of reward system function has been made by selective examinations of the functionally different VTA-NAcc dopamine neuron populations [showing, for instance, that a projection activated by rewards, but not aversive signals, mediates fear extinction learning through phasic dopamine release at the time when the CS is unexpectedly not followed by the US (96), in line with the conceptualization of fear extinction as a relief-driven appetitive learning process (352)]. In CSD, a specifically reward-driven population also phasically activates in resilient mice when they fight back, whereas these neurons deactivate specifically in non-resilient mice when they are attacked by the resident and activate when the attack ends (344). The same population is also known to be inhibited by aversive stimuli. This suggests that attacks by the resident have aversive and their termination has rewarding (relieving) character for non-resilient animals, whereas fighting the resident is associated with reward for resilient animals. This differential firing pattern persists into the social interaction test, where it is observed during phases of proximity with the test animal from the resident’s strain (344).

This apparent neural manifestation of individual differences in threat appraisal, where more positive appraisal relates to resilience (cf. sect. 2.2, FIGURE 3*B*), is causal for resilient outcomes, as shown by targeted optogenetic stimulation of the neurons, either randomly during defeat sessions or specifically at the onset of naturally occurring fighting-back sequences. Both types of stimulation increase fighting and resident-facing vigilance behaviors during the sessions as well as social interaction during later testing (and also reduce CSD-induced anxiety in other tests, indicating a globally protective function) (344).

Next to the more positive appraisal of, and the concomitantly relatively reduced stress reaction to, actual stressor encounters, registering stressor termination and quickly ending unnecessary resource expenditure through quick stress response termination (stress recovery) is considered another pathway by which positive appraisal leads to optimal stress response regulation (see sect. 2). Stressor termination only is a safety signal when it reliably predicts an extended phase of stressor absence and thus gives the possibility to replenish resources and prepare for potential future encounters. Whereas offsets of resident proximity phases in the CSD paradigm, where the aggressor approaches the intruder mouse multiply and unpredictably during a defeat session, do not function as reliable safety signals, offset of a longer stressor (such as 2–3 h of restraint) and transfer to a different context leads animals to even assign rewarding value to the stressor termination, as is evident from the development of place preference to the new context (353). The magnitude of this appetitive relief behavior after restraint negatively predicts the development of anhedonic, but not anxiety-like, behavior after a 2-wk chronic restraint procedure. This is in congruence with the human data reviewed in sect. 4.1.3, which suggest that good stressor recovery is an RM protecting against depressive symptoms. Place preference and resilience to anhedonia in this paradigm are dependent on activity of VTA-NAcc dopaminergic projections, dopamine release in NAcc, and activation of dopamine 1 (D1) receptors on NAcc MSNs shortly after stressor termination (353).

Combined, these data strongly link positive stressor appraisal in rodents with resilience and suggest that positive appraisal of stressors is associated with more activation/less inhibition of the mesolimbic reward system (344), whereas positive appraisal of stressor termination is carried by stronger phasic activation of this system (353). The latter allows classification of reward detection and exploitation in the VTA-NAcc dopaminergic system as a neural RM with protective function against anhedonic-depressive behavior, as postulated in sect. 5.6 (FIGURE 6). It still remains open whether the better function of the reward system in resilient animals results from intrinsic mechanisms that make the system more resistant to stressor-induced functional impairments or whether a lesser inhibition by the aversive system (or some other extrinsic factor) is the ultimate causal factor.

One hint comes from the observation that activation of D1-MSNs facilitates activation of the VTA-NAcc dopaminergic pathway through inhibition of inhibitory GABAergic neurons in the VTA (354). Excitatory input onto D1-MSNs is decreased after CSD in non-resilient mice, and stimulating these neurons reverses the non-resilient phenotype, suggesting that an intrinsic excitatory loop in the reward system enhances resilience (355). In this context, findings that resilient mice show long-term accumulation of ΔFosB (see also sect. 6.2) in D1-MSNs after CSD (whereas non-resilient mice show accumulation in the other MSN subtype, D2-MSNs) (356) and that raising ΔFosB levels in these cells generates resilient outcomes (357) indicate that the described induction of ΔFosB in D1-MSNs by stressors and rewards (358) is a molecular RM. ΔFosB has target genes such as the GLUA2 subunit of the AMPA-type glutamate receptor and the activity-signaling protein kinase CaMKIIα and may thus control plasticity at NAcc glutamatergic synapses (358). This permits us to postulate that ΔFosB accumulation during or after stressor exposure has long-term protective effects because it lastingly impacts the function of the reward system and thereby stress response regulation.

### 6.5. Spotlight on Neuroplasticity

A recurrent theme in our review of neurobiological RFs and RMs in animal models is the detection of neuroplasticity, induced by positive or stimulating experiences (e.g., communal nesting, enrichment, single positive events) or stressors (including controllable or moderate stressors, as in stress inoculation), and the lasting protective effect this has on future stressor encounters. This indicates that an individual capacity for neuroplasticity in circuits encoding and retrieving safe and rewarding experiences is the neurobiological basis for the individual capacity for adaptive long-term change (for the occurrence of RPs) that we postulate in sect. 1.3 to presumably be a central RF.

**FIGURE 9.**
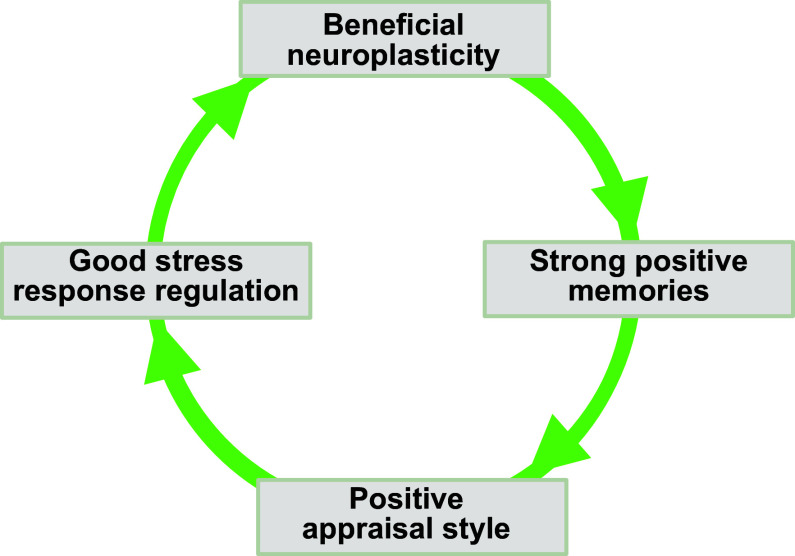
Neuroplasticity hypothesis of resilience. A virtuous cycle of good stress response regulation, high beneficial neuroplasticity, strong positive (safety and reward) memories, and positive appraisal style.

Neuroplasticity, a fundamental mechanism of neural adaptation to changing environmental demands, is impaired in mood disorders and many animal models of stress (321), and acute stress generally impairs the flexible incorporation of new experiences into existing schemata (54) and specifically also the encoding of safety memories (55). Individuals with stress-related dysfunctions not only show stronger acute stress reactions but also memory biases in favor of negative and against positive contents, which in turn may underlie their negative appraisal biases and worse stress response regulation (sect. 4.1.3). From a resilience perspective, this suggests the opposite reciprocal relationships in resilient individuals, whereby better stress response regulation (sect. 2.2, FIGURE 2*A*) facilitates beneficial forms of neuroplasticity resulting in stronger positive (safety and reward) memories, whereas stronger positive memories and the resulting more positive appraisals of stressful situations in turn facilitate stress response regulation. This could be called the virtuous cycle of resilience (FIGURE 9). The current data now permit us to link this general memory-based idea (30) with concrete neuroplastic processes (e.g., adult neurogenesis, BDNF, ΔFosB expression) in defined neural circuits or brain regions/systems (e.g., hippocampus/dentate gyrus, mPFC, reward system, hippocampus-NAcc projections, mPFC-subcortical projections) and to postulate a general positive neuroplasticity hypothesis of resilience.

Clearly, neurobiological resilience research will discover further resilience-promoting plasticity mechanisms, including their potential genetic and epigenetic bases (e.g., Refs. 28, 359–363), and it will find new ways to boost them, whether acutely [such as with drugs like ketamine (sect. 6.2), antidepressants (364–366), or anti-inflammatory agents (367), dietary manipulations (e.g., Refs. 366, 368, 369), or noninvasive brain stimulation or neurofeedback procedures (370)] or also in the longer term with suitable stress inoculation or earlier-life procedures.

### 6.6. HPA Axis

We have focused the neurobiological part of this review on brain regions and systems that traditionally are not considered part of the aversive system of the brain (comprising, e.g., amygdala, bed nucleus of the tria terminalis, anterior insula, dACC, or brain stem effector regions including the central output stations for the SAM and HPA systems). Hippocampus, PFC, and the reward system contribute to stress response regulation indirectly by “supporting” the aversive system with auxiliary functions (cf. sect. 3.3 and TABLE 1). We are aware of only one observation that may tentatively locate a neurobiological RM at the level of the aversive system, based on the finding in the endocrine literature of a cross-sectional association with depression of a relative failure to suppress CORT blood levels by administration of the synthetic CORT derivative dexamethasone, alone or in combination with corticotropin-releasing hormone/factor (139). This indicates a disturbed negative feedback regulation of the HPA axis at the level of the anterior pituitary (84) in depression. There are also indications that suboptimal HPA axis regulation may have a genetic basis and be influenced by environmental factors (362). However, no studies controlling for stressor exposure and no prospective data are available. We can therefore only speculate that individuals resilient specifically to generalized anxiety/depression-type symptomatology, as in major depression, may have better HPA axis feedback regulation, thus being less likely to show extreme or long-lasting HPA axis overactivation (hypercortisolism) and its eventual blunting (hypocortisolism) (sect. 4.1.3 and introduction to sect. 6).

In the remainder of this review, we focus on asking whether the proresilience functions of the hippocampus, the PFC, and the reward system in these brain regions or systems may also be supported by RMs in the body. Data are available for the immune system, the gut, and the barriers that separate gut and blood and blood and brain.

## 7. SYSTEMS BIOLOGICAL RESILIENCE MECHANISMS

### 7.1. Immune System and Blood-Brain Barrier

Meta-analyses and large-scale studies cross-sectionally link elevated blood levels of proinflammatory cytokines such as C-reactive protein (CRP), tumor necrosis factor alpha (TNF-α), IL-6, and IL-12 to acute depression, indicating a systemic inflammatory status in the patients (371–373). Meta-analyses also indicate potentially elevated IL-6 concentrations in cerebrospinal fluid (CSF) of depressed patients (374). In PTSD, too, there is evidence for elevated markers of peripheral inflammation (375, 376). In depression, CRP levels, which can be used as representative marker of peripheral inflammation, in particular are also found to be elevated even when statistically controlling for major stressors such as childhood trauma, low socioeconomic status, or ill physical health (371, 373); in PTSD, the same is observed when controlling for PTE exposure (377). This suggests that peripheral, and perhaps also central, inflammation is not merely an irrelevant by-product of exposure but is related to the pathophysiology. These findings are mirrored by a mouse study showing higher proinflammatory cytokine levels in non-resilient mice after CSD, whereas resilient mice had higher levels of the anti-inflammatory cytokine IL-10 (378).

In depression, there is good evidence that peripheral inflammation is a cause, rather than a consequence, of the disorder. This is suggested by Mendelian randomization analysis (379), by observations that chronic immune therapies for cancer or hepatitis frequently induce depression (e.g., Ref. 380), by observations that patients with immune/inflammatory illnesses including cardiovascular disease, irritable bowel disease, rheumatoid arthritis, and several autoimmune conditions often exhibit depressive and anxious symptoms (381–383), which in turn can be treated with anti-inflammatory agents (384, 385), as well as by evidence for successful treatment of depressive symptoms specifically in depressed patients with high CRP baseline levels (386, 387). Nevertheless, it has not been possible so far to conclusively demonstrate a prospective relationship between inflammation and depression or PTSD in longitudinal epidemiological studies because of conflicting results (388–390). Most of the meta-analyzed studies did not control for stressor exposure.

Of particular value, therefore, one study in *N* = 518 biologically male young adults identifying as sexual or gender minorities found that IL-1β levels positively interacted with subsequent PTE exposure over a 1-yr period in predicting pre-post changes in depression symptom severity, including when controlling for baseline exposure (391). A composite inflammatory index reflecting several cytokines showed analogous effects on perceived stress. A study in *N* = 1,719 male soldiers reported that baseline CRP blood concentrations collected before a 7-mo war zone deployment predicted PTSD symptom severity 3 mo after the deployment, while controlling for the independent effects of PTE exposure and baseline symptoms (392). A final prospective study investigated blood leukocyte mRNA levels 1 mo before and 3 mo after war zone deployment in *N* = 47 versus 47 exposure-matched male soldiers who were initially healthy and presented either with or without PTSD 3 mo after deployment (29). PTSD development was linked with higher expression of coregulated genes related to the innate immune response and IFN signaling before deployment. A similar expression pattern was found in a replication sample of *N* = 24 versus 24 soldiers. These data indicate that preexisting inflammation is a risk factor for stress-related dysfunctions. This conclusion is also consistent with a well-powered survivor study in *N* = 684 mainly male soldiers, where both antibody-stimulated T cell cytokine production and lipopolysaccharide (LPS)-induced monocyte cytokine production in incubated blood, drawn 1 mo after a 4-mo deployment, interacted with PTE exposure during deployment and life event exposure in the first year after deployment in predicting higher PTSD symptom development in the two postdeployment years (393). The study also controlled for early-life PTEs and medication.

In concordance with the human findings, animal studies using the CSD paradigm have observed indicators of inflammation in non-resilient mice already shortly after the defeat (in females only: Ref. 394) and, more importantly, before defeat (in males, no female mice included: Ref. 395). Most strikingly, in the former study prolonged treatment with anti-inflammatory phytochemicals before defeat enhanced social interaction at the later test, whereas in the latter study manipulations to enhance and inhibit IL-6 production before defeat inhibited and enhanced, respectively, later social interaction. There were no effects on anxiety-like behavior. In another causal manipulation study, inhibiting upregulation by CSD of a stress-responsive microRNA cluster in monocytes also enhanced social interaction at test (396). These animal studies support the idea that good immune system regulation, perhaps via anti-inflammatory mechanisms related to IL-10 production or also involving the anti-inflammatory actions of CORT (84), is an RF, and they suggest potential for anti-inflammatory interventions in promoting resilience.

Interestingly, levels of inflammatory markers in depression are specifically associated with anhedonic and amotivational symptoms including loss of appetite, energy, and interest in doing things (371, 397), symptoms that are also typically observed after chronic immune therapy (397), where they go along with reduced reward responsiveness and aberrant dopamine metabolism in the ventral striatum (398). In PTSD, CRP levels may be most strongly associated with avoidance and fatigue, rather than with arousal symptoms (148, 399), that is, with too little rather than too much of behavioral activation and motivational drive. These symptoms mirror the sickness behavior that is observed in rodents after infection or experimental immune challenges and that presumably serves to help the organism conserve energy and recuperate (299, 400). The association between inflammation and motivation can also explain why an anti-inflammatory treatment of depressed patients had its strongest effects on symptoms of amotivation (387). This general picture is in line with the observation that relationships between inflammation markers and neuroimaging indexes in healthy people and patients are most consistently observed in reward system areas (397). These findings do not exclude other effect pathways of inflammation, notably altered hippocampus (see, e.g., Ref. 401), amygdala (e.g., Ref. 397), or PFC (e.g., Ref. 402) function.

One possible cause for the peripheral inflammatory states that compromise reward system function and make individuals vulnerable to stress-related anhedonia is prior chronic stressor exposure itself (besides injury, infection, or poisoning). This is indicated by associations with inflammation cross-sectionally of low socioeconomic status (403), exposure to terror risks (in women; Ref. 404), and PTE exposure more generally (405) and prospectively of social isolation (406) and, most impressively, some forms of childhood adversity, where the heightened inflammatory status can still be observed years later in adulthood (373, 407–409). Causation by exposure is also in line with evidence for immune activation in animal models of chronic stress (301, 382, 400). First evidence for the full causal chain from adversity to depression via inflammation was recently also provided by a cross-sectional mediation analysis in depressed patients with different levels of life event exposure and peripheral inflammation (410). It is unclear to what extent, and how, hyper- or hypocortisolism contributes to stressor-induced immune activation (411). However, autonomic imbalance (overactivity of the SAM relative to the parasympathetic nervous system, as manifest in low heart rate variability) is both cross-sectionally and prospectively associated with inflammation (412), providing another potential link between stressed states and stress-related mental dysfunction via inflammation.

Together, these findings strongly suggest an important role for inflammation induced by stressors or from other sources in compromising the function of the reward system, producing reduced reward-driven activation, and more generally in reducing energy expenditure and motivational drive, eventually giving rise to full depression-type symptomatology when further stressors hit. Although largely untested, resilience might involve brakes to these processes at different levels of the chain, including at the level of immune system activation in blood, bone marrow, spleen, and gut and its modulation by the SAM and HPA systems and vagal activity (293, 382, 413, 414); the level of transmission of peripheral inflammation signals to the brain via the BBB (299, 301) and through the vagus (415); and the level of activation of immune cells in the brain (microglia) and cytokine-induced shifts from production of serotonin to potentially neurotoxic kynurenine (299, 301).

**FIGURE 10.**
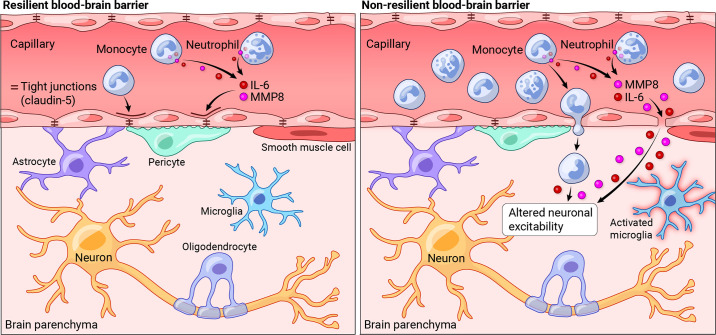
Simplified model of blood-brain barrier (BBB) function in resilience. Besides endothelial cells, the neurovascular units contain several cell types that together protect and regulate neuronal function. Resilient mice have stronger levels of the tight junction protein claudin-5 in nucleus accumbens (NAcc) (males) and prefrontal cortex (PFC) (females), making the BBB less permeable to inflammation factors (e.g., IL-6, MMP8) and activated immune cells and preventing consequential parenchymal microglia activation and changes in neuronal excitability during states of systemic inflammation. Image is copyright of Mount Sinai Health Systems and used with permission.

A particularly promising vantage point is the function of the BBB and the broader neurovascular unit that, besides endothelial cells, also contains surrounding pericytes, smooth muscle cells, astrocytes, microglia, and oligodendrocytes, which together regulate cerebrovascular function (416) (FIGURE 10). Depression is associated with increased risk of cardiovascular morbidity and mortality (417, 418), and chronic inflammation and sustained increases in circulating proinflammatory cytokines have been associated with atherosclerotic plaque formation, progression, and rupture (419) and breakdown of endothelial and epithelial barriers in several peripheral and central tissues (382). These general links between depression, inflammation, and vascular function are reflected in meta-analytic evidence for increased BBB permeability in depression, which may relate to the increased CSF levels of IL-6 in this disorder (374). This is also in congruence with animal studies linking inflammation, depression-like behavior after stressor exposure, and BBB hyperpermeability in hippocampus, NAcc, and PFC (295, 296, 420–422).

Male resilient mice after CSD show higher protein levels of claudin-5 in the hippocampus than non-resilient and nonstressed control mice (296). Claudin-5 is an endothelial tight junction protein with a key role in assuring BBB impermeability. When the *CLDN5* gene is knocked out in the hippocampus or NAcc before defeat, social interaction at test is reduced, and other depressive-like, but not anxiety-like, behaviors are also enhanced, along with higher IL-6 levels in the parenchyma. Rescue of *CLDN5* expression after defeat in knockout mice rescues social interaction (296). Similar observations were later made for the PFC of female mice (295). These data indicate a protective role of claudin-5 for depression-like consequences of stressor exposure. It is not clear from these experiments whether higher claudin-5 levels in resilient mice predate stressor exposure (whether claudin-5 is a molecular RF) or whether it develops during or after exposure and thereby contributes to the resilient outcome (whether claudin-5 upregulation is a molecular RP).

Another CSD study used a more fine-grained phenotyping of resilient stressed mice into a group that showed high social interaction with a member of the resident’s strain and another group that showed high social interaction with a conspecific from a different strain than the resident’s (aggressors’) strain, but not with the member of the resident strain (see sect. 8.1), a phenotype reminiscent of the intact threat-safety discrimination in resilient humans (TABLE 1). This study found a pattern of gene expression in neurovascular unit cells after social defeat specifically in the discriminating mice that was consistent with negative regulation of the mTOR (mechanistic target of rapamycin) pathway (423), which is also activated in the peripheral immune system of stressed mice and humans exhibiting inflammation (424). Accordingly, systemic administration of the mTOR inhibitor rapamycin before CSD prevented defeat-related reductions of social interaction, implicating another molecular pathway in resilience (423). A specific relation to BBB or immune system function was not demonstrated.

A final study found different behavior of the arterial cerebrovasculature to dilatory and constricting stimulation in mice resilient to the anhedonic effects of chronic restraint stress, relative to non-resilient and nonstressed mice (425), further highlighting a possible implication of this brain-body interface.

Importantly also, these conclusions imply that limiting inflammation and/or BBB dysfunction in exposed individuals may be a promising prevention strategy. In this context, the observation that a psycho-social intervention dampens the association between low socioeconomic status in early life and inflammation in adulthood (426) indicates that the known anti-inflammatory effects of psycho-social interventions (427, 428) may effectively provide protection against stressor-induced inflammation and, by extension, mental dysfunction. Other routes, pharmacological (e.g., Refs. 385, 429, 430), dietary (e.g., Ref. 368), lifestyle dependent (431), or influencing metabolism (432, 433), are conceivable.

### 7.2. Gastrointestinal System and Microbiota

From large cross-sectional datasets, there is evidence for associations between depression and general gut microbiota composition, certain bacterial taxa, enterotypes, and microbial metabolic pathways (434–436). The most consistent finding in these studies is that depressed patients generally diverge in their microbiota composition from healthy control subjects and that they have a reduced abundance of the *Coprococcus* taxon. *Coprococcus* bacteria produce the short-chain fatty acid (SCFA) butyrate, which protects the gut barrier to the circulation and potentially also the blood-brain barrier and has anti-inflammatory properties (437, 438) and may also be involved in dopamine metabolism (436). Cross-sectional analyses also indicate associations between PTSD and certain taxa in the gut (439). Moreover, there is also emerging evidence that dietary manipulations targeting the microbiome can alleviate depression (e.g., Refs. 440, 441) and perceived stress (e.g., Refs. 442, 443), going along with reductions in peripheral inflammation markers (444).

These observations and associations between gastrointestinal disorders and depression (301) indicate a causal role for disturbances in the microbiome and, more generally, in gut function in the development of stress-related mental impairments. Effects of disease may partly pass via increased release of proinflammatory bacterial products and the increased passage of such products, or also microbes, through an impaired gut barrier; other effect pathways may involve reduced intestinal serotonin production (301, 382, 445). Gut function, in turn, is sensitive to stressors and inflammation (301, 382, 446).

With particular relevance for resilience, one study in male and female healthy students in preparation for an important exam showed that regular consumption of fermented milk containing the *Lactobacillus casei* strain Shirota (*N* = 24) relative to consumption of a placebo milk preparation (*N* = 25) over 8 wk in the run-up to the exam reduced exam-related perceived stress, though not anxious or depressive symptoms (447). Another study with an 11-wk administration of the same formulations in a similar cohort (*N* = 48 vs. 46 students) showed a beneficial effect on several measures of exam-related sleep disturbances (and again not on anxiety or depression) (448).

By contrast, intake of the *Lactobacillus gasseri* strain CP2305, heat-inactivated in tablet form, compared with placebo was shown to reduce not only exam-related sleep disturbances but also anxiety and depression (*N* = 31 vs. 29) (449). These positive effects were accompanied by mitigation of stress-induced changes in fecal microbiota and in the fecal concentration of the SCFA n-valeric acid, another microbial metabolite. The study followed upon an earlier study where the inactivated strain was administered to the students in a daily drink over 12 wk (*N* = 24 vs. 35) and reduced only exam-related sleep disturbances, accompanied by normalization of defecation frequency and daily fecal output (450).

A very small study showed a relieving effect of 2 wk of intake of tablets with the butyrate-producing *Clostridium butyricum* on anxiety in anticipation of surgery in *N* = 10 versus 10 cancer patients (451).

**FIGURE 11.**
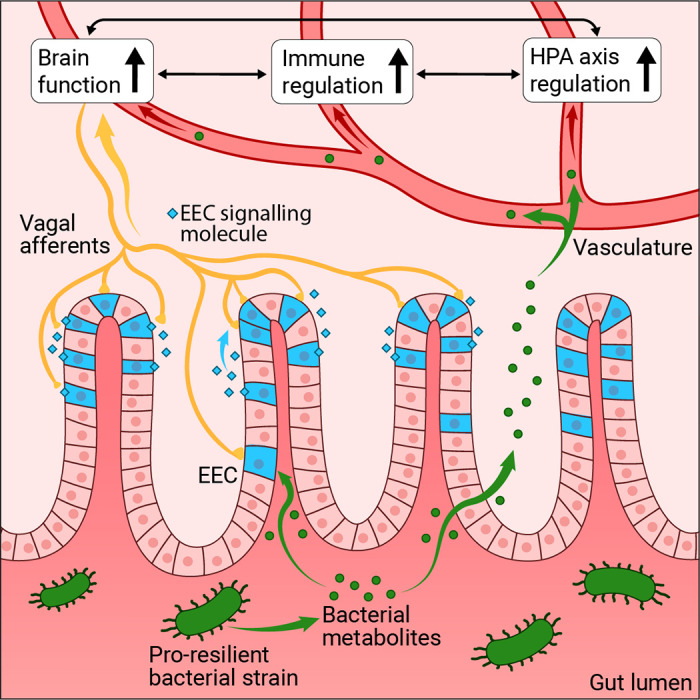
Simplified model of gut microbiome influences of resilience. Proresilient bacterial strains release metabolites (e.g., anti-inflammatory short-chain fatty acids) that can protect gut barrier integrity and also permeate the epithelium and enter the vasculature. Bacterial metabolites can also stimulate enteroendocrine (EEC) cells in the epithelium to activate vagal afferents to the brain via direct contacts or the release of signaling molecules. Combined, these humoral and neural effectors promote immune and hypothalamus-pituitary-adrenal gland (HPA) axis regulation and protect brain function (e.g., in the hippocampus). Image is copyright of Mount Sinai Health Systems and used with permission.

Together, these findings indicate that a specific composition of the gut microbiome, potentially even specific strains, may convey some extent of resilience to stressors, presumably via the production of anti-inflammatory and barrier-protective SCFAs or some other way of providing immunoregulation (452). In support of this, in male rodents subcutaneous administration of *Mycobacterium vaccae* NCTC 11659 before or during exposure to chronic social subordination inhibits the development of anxiety-like and socially avoidant behavior, an effect that depends on recruitment of anti-inflammatory regulatory T cells (453, 454). *M. vaccae* administration also reduces the stressor-induced responsivity of hippocampal microglia to an ex vivo immune challenge and has been shown to have other local anti-inflammatory actions (455). Generally, there is a close link between microbiome composition and both hippocampus-dependent learning and memory functions and adult hippocampal neurogenesis, which in addition to immuno-metabolic mechanisms may also involve modulation of vagal and HPA axis activity (456, 457) (FIGURE 11).

It is unclear yet what characterizes proresilience bacterial strains, but one hypothesis is that these are microbes that are transmitted by mothers or other family members during development or by organisms present in natural (rural) environments or also old pathogens from the hunter-gatherer phase of human evolution, to which humans have developed tolerance (452). In support specifically of a beneficial influence of the presence of farm animals during upbringing, one study showed that young male adults raised in a rural versus urban environment (*N* = 20 vs. 20) showed relatively better immune system regulation in response to an acute stressor (458).

### 7.3. Summary of Systems Biological Findings

**FIGURE 12.**
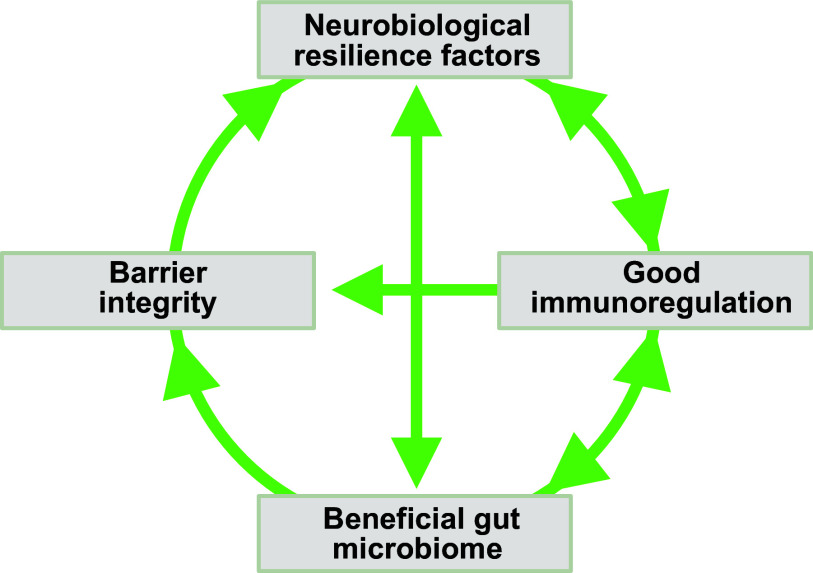
Positive interactions between neurobiological resilience factors, good immunoregulation, a beneficial gut microbiome composition, and gut barrier and blood-brain barrier integrity.

The reviewed literature indicates that resilience-relevant functions of the brain, notably in the hippocampus, the PFC, and the reward system, benefit from good immunoregulation, a certain gut microbiome composition, and integrity of the gut and blood-brain barriers. These peripheral RFs may be particularly useful in the protection against the more severe stress-related impairments on the depression/generalized anxiety/severe PTSD side of the spectrum, connected with dysfunctions of the reward system (TABLE 1, FIGURE 6). It is unclear to what extent these RFs specifically benefit neuroplasticity in these areas. The apparent reciprocal relationships between stress and immune and gastrointestinal function suggest negative potentiating interactions underlying the development of stress-related dysfunctions and positive interactions, similar to the vicious cycle in the case of neuroplasticity (sect. 6.5), underlying resilience (FIGURE 12).

## 8. OUTLOOK

In this review, we have integrated the human and animal literature on stress resilience from a biological angle, with the intention not only to obtain a better mechanistic understanding of the phenomenon but also to find targets for the development of new, biologically informed preventions. Promising anchor points have been highlighted throughout this review.

We have emphasized that human resilience research needs more well-powered prospective-longitudinal studies with careful control for individual stressor exposure (for methodological recommendations, see Ref. 27) and also that there is a dearth of studies testing causal manipulations of RFs or RMs. Exposure-controlled studies, in particular, will also allow us to better address questions of sex- or gender-related differences in resilience.

We close this review with a discussion of two topics we believe have specific importance for the future development of biological resilience research. First, we address methodological questions related to translational research in animals, which this review has shown is a key source of insights. Despite its obvious utility, we believe the field is ripe for considerable advancement and refinement of its methodological approaches. Second, we provide some initial thoughts on what could be the ethical and societal implications of introducing biologically informed (especially pharmacological or dietary) resilience-promoting interventions.

### 8.1. Methodological Questions in Animal Research

#### 8.1.1. Validity.

**FIGURE 13.**
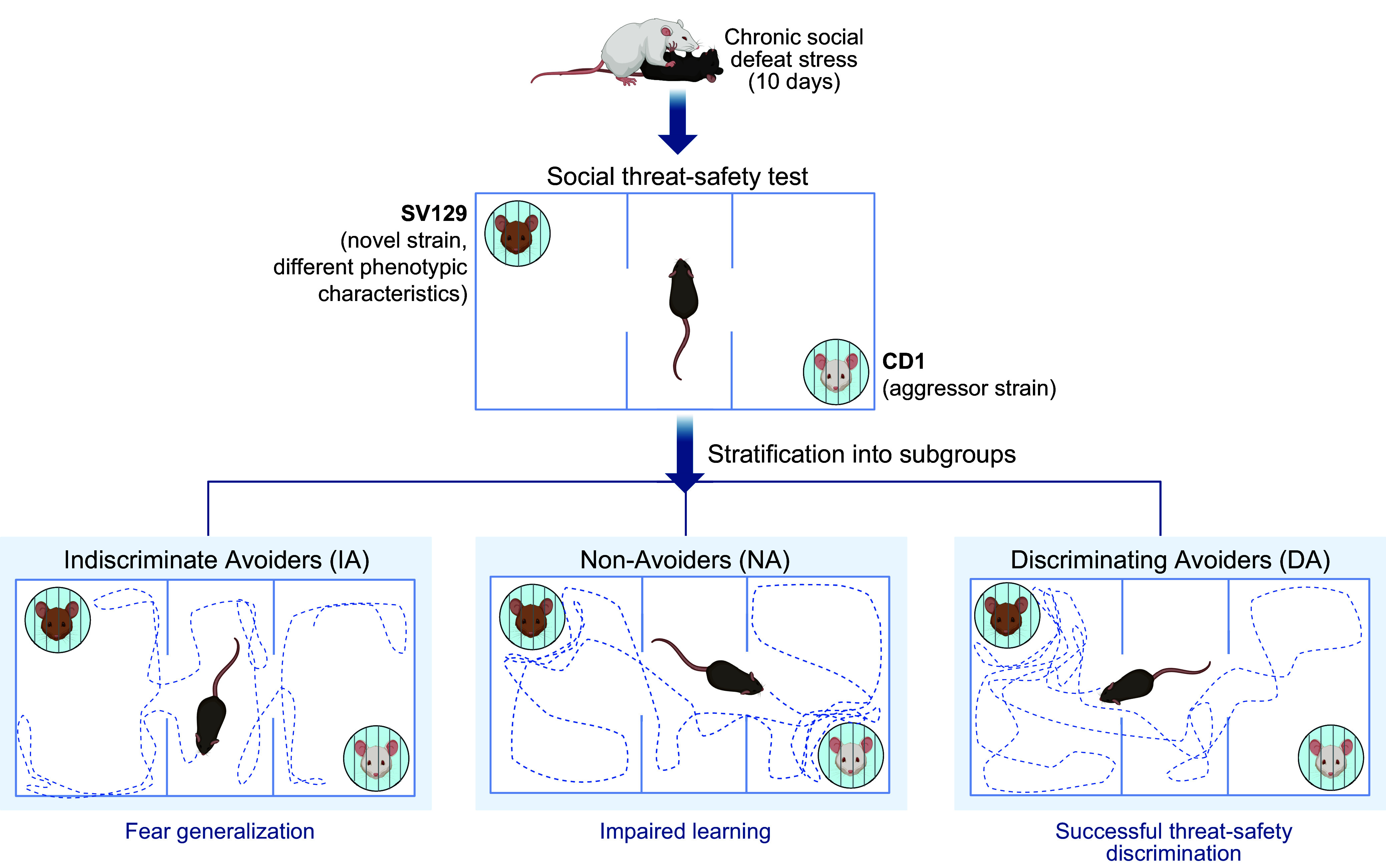
Social threat-safety test. *Top*: in this post-CSD (chronic social defeat) test, the defeated test mouse can choose to interact with a mouse from the same strain as the residents (aggressors) during CSD (here: CD1) or a mouse from a phenotypically different strain (here: SV129). *Bottom*: a subgroup of test mice will interact with the other mouse to similar levels as nonstressed control mice but avoid the resident strain (*right*, “Discriminating Avoiders”). Other subgroups will avoid both strains (*left*, “Indiscriminate Avoiders”) or strongly interact with both (*center*, “Non-Avoiders”).

CSD can be understood as an assault on brain functions supporting appetitively motivated social behavior, leading to generalized impairments in social interaction. This interpretation is, however, not in agreement with the observation that CSD-induced social avoidance is on average specific toward mice from the residents’ (aggressors’) strain and does not generalize to other mouse strains, which intruder (test) mice continue to approach. This has been shown in the social threat-safety test (459, 460), where the test mouse can freely choose between a mouse from the resident strain and a mouse from another, neutral strain with different phenotypic characteristics (e.g., fur color) (FIGURE 13).

If CSD-induced social avoidance is indeed dependent on the phenotypic characteristics of the social target (460), it cannot be a general social deficit but is a learned (socially conditioned) behavior. This is also supported by the finding that CSD-induced social avoidance can be reversed by extinction training, where the test mouse repeatedly experiences nonharmful social confrontations with mice from the resident strain (460).

These insights have important consequences for the use of the CSD + social interaction model in resilience research. A detailed analysis of the social interaction data obtained in the social threat-safety test reveals three different subgroups within the pool of defeated mice. In addition to the prototypical group of “Discriminating Avoiders,” there are animals that avoid both strains (“Indiscriminate Avoiders”) and animals that avoid none (“Non-Avoiders”) (FIGURE 13). Comparison with data from the classical social interaction test in the same animals shows that the Discriminating Avoiders are “hidden” in the group of avoidant animals that are classified as non-resilient according to the established stratification criterion, as they avoid the resident strain (459). This may be problematic, since the good threat-safety discrimination shown by these animals is also a hallmark of resilience in humans (sect. 4.1.1, TABLE 1). Conversely, the nonavoiding phenotype, which is classified as resilient via the classical social interaction test, exhibits deficits in conditioning also to nonsocial threat cues. One possible source of this broad fear conditioning impairment in these animals may be a general learning impairment; another may be that these animals do not appraise the aggressor as a threat in the first place (344) (see sect. 6.4). On this basis, it has been suggested that the “resilient” animals according to the classical stratification may be cases of extreme resilience (423) or even of maladaptive behavior (459), and a focus on Discriminating Avoiders as a clearly resilient phenotype has been proposed. The new triadic behavioral stratification using the social threat-safety test is paralleled by subgroup-specific transcriptional signatures in fear- and emotion-related brain areas (423, 459). Hence, translational resilience research may benefit from a more fine-grained behavioral analysis.

#### 8.1.2. High-resolution behavior monitoring and data-driven analysis.

The same theme emerges from recent advances in the measurement and quantification of animal behavior under observer-independent conditions. These new technologies make it possible to go beyond defining behavioral phenotypes with single indexes derived from artificial behavior tests applied at defined time points in a study (e.g., a social interaction test) and instead rely on the longitudinal, temporally highly resolved monitoring of animal behavior and physiology over longer time periods and on machine learning-based unsupervised data analysis (461, 462), to thus break down behavior into quantifiable subcategories and even smaller units, syllables, or motifs (463–465). Exploiting the richness of behavioral variables provided by this approach, researchers have recently detected differences in how resilient and non-resilient mice behave toward the resident in the CSD paradigm and have been able to link these behaviors to simultaneous, temporally resolved recordings of dopamine system activity and even to use real-time automated behavior analysis to time optogenetic manipulations of the system to specific behaviors (344). This has led to groundbreaking insights into how neural organization of behavior impacts resilience (sect. 6.5). We can thus realistically expect that the next years will see a rapid improvement in our possibilities to identify, decode, and promote resilience in experimental animals.

#### 8.1.3. Process identification through longitudinal monitoring.

Current stratification approaches use a single outcome test to assess resilience, and measurements of neurobiological correlates of resilience are frequently performed at a single time point in a study, for instance when animals are euthanized after the outcome test. These approaches fall short of taking into account the dynamic nature of resilience, where mental health problems during and after stressor exposure can take different time courses (FIGURE 1*A*) and RFs may change in strength or efficacy over time in processes of adaptive change (RPs; FIGURE 1*C*). Human resilience research increasingly emphasizes the necessity to repeatedly monitor stressors, mental health changes, and potential RFs (27) to describe RPs. Recent animal studies have also used repeated-measurement approaches (e.g., Refs. 466, 467), and we expect that the increasing availability of observer-independent longitudinal monitoring technologies, discussed above, will also greatly benefit the process-based study of resilience.

### 8.2. Potential Ethical and Societal Implications of Biological Resilience Promotion

Known psycho-social interventions aiming at preventing stress-related mental health problems are characterized by small or absent effects, and long-term effects are unclear (e.g., Ref. 468). Psycho-social interventions also have the disadvantage that they are often less accepted and sought specifically by men, presumably because of perceived gender role conflicts (469). On this basis, the individual and societal (e.g., Ref. 470) costs associated with stress-related mental dysfunctions combined with the sheer prevalence of stress disorders (471) appear to justify a search for improved solutions (472).

It is likely that biological resilience research will come up with new strategies for prevention. These may include direct manipulations of brain function, such as with neuropharmacological or neurostimulation or neurofeedback methods (see sect. 6.5), but will in the first place probably involve pharmacological or dietary manipulations of peripheral functions with indirect action on the brain, leveraging the growing insights of systems biology (see sect. 7). The latter approaches may be safer and less costly and thus more applicable to (larger) groups of individuals who are not yet patients but have a disease risk. An attractive solution may be to add a biological onto a psycho-social intervention, to reach bidirectional-synergistic effects, where biological changes support psychological improvement and psychological improvements support better biological function. If a biological intervention element as part of a combination treatment indeed enhanced intervention acceptance among men (473) or generally among persons skeptical toward psychological treatments, this might even allow some of these individuals to experience the beneficial effects of psycho-social interventions for the first time. Finally, a safe and evidence-based pharmacological or dietary intervention may also be preferable to the frequent practice of self-medication for stress management (alcohol, tobacco, cannabis, stimulants) (474).

These potential benefits have to be weighed against potential risks. First, the risk of medical side effects coming with biological interventions is a challenge to the principle of nonmaleficence, especially when interventions are administered to nonpatients in the service of prevention (475). Biological interventions may also have the undesirable consequence of making it easier for users to avoid confrontation with personal or interpersonal problems as well as to avoid the use of active coping that could otherwise help positively transform harmful environmental constellations or reduce external demands. There is also a risk that an effective drug or other treatment might be misused for self-optimization (476) rather than for disease prevention. Finally, the existence of a biological solution might lead to pressure on stressed individuals by third parties (e.g., employers, educators, families, friends) to rely on such a solution and allow these parties to circumvent measures of structural demand reduction.

Risks of individual problem avoidance or self-optimization may be partly countered by combination treatment, but it can of course not be excluded that effective biological solutions might become available outside a combined setting. Furthermore, even psycho-social interventions might promote problem avoidance or self-optimization, in particular when they exclusively focus on individual change and/or cognitive coping. The risk of external pressure is also not specific to biological solutions. These latter considerations highlight that potential ethical-societal problems of resilience-promoting interventions are not necessarily restricted to biological approaches. Rather, a general discussion of the further implications of resilience research that must include relevant stakeholder groups is warranted, and ethical guidelines should be developed. These must also address exit strategies, that is, decision-making criteria for intervention discontinuation in individuals whose level of disease risk has decreased (477). The present discussion can only provide initial keywords for such endeavor.

### 8.3. Conclusion

In summary, we anticipate major progress in biological resilience research from recent advances in human and translational systems and neurobiology. This will allow testing of a preliminary working model of resilience that integrates specific functions and neuroplasticity of key brain areas and circuits, namely the hippocampal, prefrontal, and reward systems, with resilience-promoting systems biology factors derived from immune modulation, gut microbiome composition, and maintenance of barrier integrity. We expect that this model will be significantly refined and extended by the discovery of further resilience mechanisms, including in other functional systems. Ultimately, an increasing biological understanding of resilience will open up new avenues for prevention.

## GRANTS

This work has received funding from the European Union’s Horizon 2020 research and innovation program under grant agreement 777084 (DynaMORE project, to R.K.) and the European Union’s Horizon Europe program under grant agreement 101057529 (FAMILY project, to R.K.). Funding was further received from the Landesinitiative Rheinland‐Pfalz and the Resilience, Adaptation, and Longevity (ReALity) initiative of the Johannes Gutenberg University of Mainz (MORE project, to M.B.M. and R.K.), National Institutes of Health Grants R01MH114882, R01MH104559, and R01MH127820 (to S.J.R.), the Boehringer Ingelheim Foundation (individual phenotyping and high-resolution automated behavioral analysis, to M.B.M), and the Leibniz ScienceCampus NanoBrain (to M.B.M.).

## DISCLAIMERS

Views and opinions expressed are those of the authors only and do not necessarily reflect those of the European Union or the European Health and Digital Executive Agency (HADEA). Neither the European Union nor the granting authorities can be held responsible for them.

## DISCLOSURES

No conflicts of interest, financial or otherwise, are declared by the authors.

## AUTHOR CONTRIBUTIONS

R.K., S.J.R., and M.B.M. prepared figures; drafted manuscript; edited and revised manuscript; and approved final version of manuscript.
